# Candidate genes and sequence variants for susceptibility to mycobacterial infection identified by whole-exome sequencing

**DOI:** 10.3389/fgene.2022.969895

**Published:** 2022-10-20

**Authors:** Alexander Varzari, Igor V. Deyneko, Gitte Hoffmann Bruun, Maja Dembic, Winfried Hofmann, Victor M. Cebotari, Sergei S. Ginda, Brage S. Andresen, Thomas Illig

**Affiliations:** ^1^ Laboratory of Human Genetics, Chiril Draganiuc Institute of Phthisiopneumology, Kishinev, Moldova; ^2^ Hannover Unified Biobank, Hannover Medical School, Hannover, Germany; ^3^ Laboratory of Functional Genomics, Timiryazev Institute of Plant Physiology Russian Academy of Sciences, Moscow, Russia; ^4^ Department of Biochemistry and Molecular Biology, University of Southern Denmark, Odense M, Denmark; ^5^ The Villum Center for Bioanalytical Sciences, University of Southern Denmark, Odense, Denmark; ^6^ Department of Clinical Genetics, Odense University Hospital, Odense, Denmark; ^7^ Department of Mathematics and Computer Science, University of Southern Denmark, Odense, Denmark; ^8^ Department of Human Genetics, Hannover Medical School, Hannover, Germany; ^9^ Municipal Hospital of Phthisiopneumology, Department of Pediatrics, Kishinev, Moldova; ^10^ Laboratory of Immunology and Allergology, Chiril Draganiuc Institute of Phthisiopneumology, Kishinev, Moldova

**Keywords:** disseminated *tuberculosis*, BCG-itis, primary immunodeficiency (PID) disorders, whole-exome sequencing (WES), trio analysis, pre-mRNA splicing, candidate genes approach, oligogenic inheritance

## Abstract

Inborn errors of immunity are known to influence susceptibility to mycobacterial infections. The aim of this study was to characterize the genetic profile of nine patients with mycobacterial infections (eight with BCGitis and one with disseminated tuberculosis) from the Republic of Moldova using whole-exome sequencing. In total, 12 variants in eight genes known to be associated with Mendelian Susceptibility to Mycobacterial Disease (MSMD) were detected in six out of nine patients examined. In particular, a novel splice site mutation c.373–2A>C in *STAT1* gene was found and functionally confirmed in a patient with disseminated tuberculosis. Trio analysis was possible for seven out of nine patients, and resulted in 23 candidate variants in 15 novel genes. Four of these genes - *GBP2*, *HEATR3*, *PPP1R9B* and *KDM6A* were further prioritized, considering their elevated expression in immune-related tissues. Compound heterozygosity was found in *GBP2* in a single patient, comprising a maternally inherited missense variant c.412G>A/p.(Ala138Thr) predicted to be deleterious and a paternally inherited intronic mutation c.1149+14T>C. Functional studies demonstrated that the intronic mutation affects splicing and the level of transcript. Finally, we analyzed pathogenicity of variant combinations in gene pairs and identified five patients with putative oligogenic inheritance. In summary, our study expands the spectrum of genetic variation contributing to susceptibility to mycobacterial infections in children and provides insight into the complex/oligogenic disease-causing mode.

## Introduction

Tuberculosis (TB) is a serious infectious disease usually caused by *Mycobacterium tuberculosis* (*M. tuberculosis*), but also by *M. africanum*, *M. bovis* and *M. canettii* species (Lawn and Zumla, 2011). About one-quarter of the world’s population has a TB infection (WHO, 2020), but most people who are infected with TB bacteria do not develop symptoms. Immunocompromised adults and young children are particularly at risk of progressing to active disease. They also develop more severe forms of TB (e.g., disseminated/milliary TB, TB meningitis). Therefore, the vaccination with attenuated *M. bovis* Bacille Calmette-Guérin (BCG) substrains is recommended to prevent severe TB disease (Roy et al., 2014). The vaccine is given routinely at birth or during childhood in countries with a high burden of TB and it is generally recognized as a safe and effective measure. Serious complications (or BCG infection) are, however, known to occur in children receiving BCG (Kourime et al., 2016).

Certain conditions may cause a child to become more vulnerable/susceptible to mycobacterial infections. It has been estimated that up to 30% of disseminated TB cases are due to monogenic/Mendelian errors (Alcaïs et al., 2005). Consistent with this estimation, several primary immunodefficiency (PID) disorders were systematically associated with mycobacterial disease (Boisson-Dupuis et al., 2015). These PIDs are heterogeneous in terms of clinical manifestations (Fekrvand et al., 2020; Tangye et al., 2020). Classical PIDs, such as combined immunodeficiency (CID) and severe combined immunodeficiency (SCID), chronic granulomatous disease (CGD) and GATA2 deficiency, are associated with increased susceptibility to diseases caused by diverse infectious pathogens, including mycobacteria. In contrast, Mendelian Susceptibility to Mycobacterial Disease (MSMD) constitutes an atypical PID caused by defects in IFN-γ-mediated immunity, leading to the selective predisposition to intermacrophagic infections, mostly *Mycobacteria* and *Salmonella* (Boisson-Dupuis et al., 2015; Bustamante, 2020). To date, over 200 disease-associated variants in 17 autosomal (*IFNGR1, IFNGR2, IL12B, IL12RB1, IL12RB2, STAT1, TYK2, IRF8, SPPL2A, IL23R, ISG15, JAK1, RORC, ZNFX1, TBX21, IFNG* and *USP18*) and two X-linked (*IKBKG* and *CYBB*) genes have been described in patients with MSMD (Bustamante, 2020; Kerner et al., 2020; Tom Le Voye et al., 2021; Martin-Fernandez et al., 2022). These, however, account for only half of the MSMD cases studied, suggesting that mutations in other genes may be involved in genetic susceptibility to MSMD (Bustamante, 2020; Tom Le Voye et al., 2021).

The use of next-generation sequencing (NGS) offers a rapid and efficient approach to find disease-causing mutations in affected individuals and to discover new disease genes (King et al., 2018). In this study, we implemented whole-exome sequencing (WES) in an attempt to identify risk genes and rare variants in a cohort of seven case-parent trios and two single pediatric cases with mycobacterial infections (BCGitis and disseminated/generalised TB) from the Republic of Moldova. The functional effect of identified candidate variants was investigated through experimental assessment of splicing.

## Material and methods

### Patient description

Nine children (six girls and three boys aged 2–24 months) with *M. tuberculosis* complex infection were recruited through the department of Paediatrics at Municipal Hospital in Chisinau, Moldova, during 2016–2018. Among these patients, eight were diagnosed with BCGitis and one with generalised *M. tuberculosis* infection. The diagnosis relied on the assessment of clinical features and for six cases was confirmed bacteriologically and/or histologically. The clinical, demographic and immunologic patient data presented in this study are based on medical records and summarized in Table 1. Immunologic values were within the reference range for most parameters studied in most patients. We note only decreased levels of IgA in patients P2 and P7 and diminished counts of B-lymphocytes (CD20^+^) in P5. These may indicate an immune system defect. However, overall clinical/immunologic phenotypes in these cases are inconsistent with those reported for BCG-effected patients with SCID or CGD (Fekrvand et al., 2020). Also, other known PIDs associated with BCG complications, such as GATA2 deficiency, which are even rarer than SCID and CGD, were excluded for the same reasons (i.e., inconsistency with the provided clinical/immunologic phenotypes), and thus a diagnosis of MSMD was suspected.

**TABLE 1:** Patients’ clinical characteristics and laboratory findings.

Patient no.	P1	P2	P3	P4	P5	P6	P7	P8	P9^a^
Sex	M	F	F	F	M	F	F	F	M
BCG vaccine	Y	Y	Y	Y	Y	Y	Y	Y	N
Age at diagnosis (months)	6	4	8	3	2	24	2	17	20
Clinical features	BCGitis: severe axillary lymphadenitis	BCGitis: severe axillary lymphadenitis	BCGitis: severe regional lymphadenitis	BCGitis: severe axillary lymphadenitis	BCGitis: severe axillary lymphadenitis	BCGitis: supraclavicular lymphadenopathy	BCGitis: severe axillary lymphadenitis	BCGitis: osteomyelitis (os sternum)	Disseminated TB
Concomitant infections	N	N	N	N	N	N	N	N	*Klebsiella pneumoniae*, *Candida* spp.^b^
Other diseases	N	Allergic dermatitis, anemia, toxic encephalopathy	N	Anemia	Anemia	N	Anemia	Perinatal encephalopathy, breath-holding spells	N
Treatment	Surgery, H, R	Surgery, H, R	Surgery, H, R	Surgery, H, R	Surgery, H, R	Surgery, H, R	Surgery, H, R	Surgery, H, R	Cm, Lfx, Cs, E, Z
Follow-up	Alive	Alive	Alive	Alive	Alive	Alive	Alive	Alive	Alive
Bacteriological confirmation	poz	neg	poz	poz	poz	poz	neg	poz	poz
Leukocytes (х 10^9^/L)	9.1	11.2	8.4	12.4	6.5	8.1	10.7	15.8	17.7
Lymphocytes, % (absolute count х 10^9^/L)	47 (4.3)	65 (7.3)	65 (5.5)	70 (8.7)	75 (4.9)	54 (4.4)	64 (6.8)	63 (10)	32 (5.7)
Monocytes, % (absolute count х 10^9^/L)	7 (0.6)	8 (0.9)	5 (0.4)	9 (1.1)	4 (0.3)	6 (0.5)	3 (0.3)	8 (1.3)	6 (1.1)
Segmented neutrophils, % (absolute count х 109/L)	41 (3.7)	20 (2.2)	24 (2)	13 (1.6)	16 (1)	32 (2.6)	24 (2.6)	20 (3.2)	49 (8.7)
Lymphocyte phenotype, % (absolute count х 10^9^/L)^c^									
CD3^+^	83 (3.5)	83 (6)	76 (4.1)	83 (7.2)	80 (3.9)	84 (3.7)	72 (4.9)	83 (8.3)	66 (3.7)
CD4^+^	57 (2.4)	61 (4.4)	42 (2.3)	59 (5.1)	48 (2.3)	53 (2.3)	52 (3.6)	55 (5.5)	51 (2.9)
CD8^+^	15 (0.6)	21 (1.5)	19 (1)	14 (1.2)	27 (1.3)	15 (0.7)	6 (0.4)	17 (1.7)	14 (0.8)
CD20^+^	9 (0.4)	4 (0.3)	7 (0.4)	4 (0.3)	1 (0.05)	6 (0.3)	7 (0.5)	10 (1)	15 (0.8)
CD16^+^	8 (0.3)	7 (0.5)	12 (0.7)	7 (0.6)	11 (0.5)	6 (0.3)	7 (0.5)	8 (0.8)	15 (0.8)
CD56^+^	3 (0.1)	2 (0.1)	5 (0.3)	3 (0.3)	3 (0.1)	2 (0.1)	NA	1 (0.1)	NA
Phagocytic number, %	62.7	60.2	46	45.2	64.2	60	65.6	67.9	71.1
Phagocytic index	6.11	6.18	6.6	4.6	4.8	7.92	6.49	11.7	4.82
Nitro-blue tetrazolium	0.1	0.1	0.1	0.14	0.14	0.2	0.15	0.17	0.15
Serum immunoglobulin levels^d^									
IgM (g/L)	0.39	0.5	0.63	0.42	0.39	0.54	0.7	0.6	0.74
IgG (g/L)	6.2	3.6	7.2	4.12	3.97	5.8	6.2	9.4	8.2
IgA (g/L)	0.27	<0.25	0.51	0.39	0.3	0.27	<0.25	0.34	0.4
IgE (IU/ml)	30	60	12	27	33	72	3	48	48
Effected parents and siblings	N	Father with primary TB complex at 12 y.o	Monozygotic twin with BCGitis	N	N	N	N	N	Brother with primary TB complex at 5 y.o^e^

Y = yes; N = no; NA, not available; H = isoniazid; R = rifampicin; Cm = capreomycin; Lfx = levofloxacin; Cs = cycloserine; E = ethambutol; Z = pyrazinamide.

^a^ No external or internal household contacts with active TB, disease were identified for P9.

^b^ Both pathogens (*Klebsiella pneumoniae* and *Candida spp*.) were detected in the same sampling of sputum as *M. tuberculosis*.

^c^ Cells were counted by fluorescence microscopy after immunostaining with fluorochrome-conjugated antibodies; at least 100 cells were counted.

^d^ Detection by ELISA, antibody test (IgM, IgG and IgE) and nephelometric method (IgA).

^e^ The diagnosis (primary TB, complex) was made shortly after TB, diagnosis in the index case (no. P9). The sibling was BCG, vaccinated with no side effects reported/detected.

Both parents were present for the interviews and all of them, except one, denied mycobacterial disease during his/her lifetime. For the only father (P2) who announced TB during childhood, the diagnosis of primary (Ghon) complex of pulmonary TB at the age 12 years has been witnessed/confirmed by the medical record. No parents were consanguineous. DNA from both parents was available for seven probands (P1-P7). Therefore, a total of seven trios and two singletons (P8 and P9) were included in the study.

Three probands were selected based on sequencing results to experimentally validate the effects of the identified variants on RNA splicing. Two of them (P6 and P9) were available for direct RNA analysis and were invited and sampled again (both at the age 3 years), while the third proband (P2) was unavailable, thus an RNA sample was collected and used from the father instead, who harbors as well the suspected genetic variant. Additionally, four healthy children (all girls aged 3 years) were selected as controls for RNA analysis and sampled by the Municipal Hospital’s pediatric team.

Written informed consent for clinical and genetic analyses was obtained from all the parents or guardians of the subjects participating in the study. This study was approved by the Ethics Committee of the Chiril Draganiuc Institute of Phthisiopneumology (Reference # CE-20.2).

### 2.2 Exome sequencing and variant detection

DNA was extracted from blood leukocytes of all patients and their parents. Exome capturing was performed using the xGen Exome Research Panel v1.0 Kit (Integrated DNA Technologies, Coralville, Iowa), and sequenced on Illumina NextSeq 500 (Illumina, San Diego, CA) with 150-bp paired-end reads. Demultiplexing/conversion of the sequencing data was done using bcl2fastq, version 2.18. The resulting fastq files were aligned to the human reference genome (GRCh37) by bwa mem, version 0.7.17, the local realignment was done using abra, version 2.05. After the variant calling by freebayes, version 1.1.0, the annotation of the resulting vcf file was done by SnpSift/SnpEff, version 4.3t, and vcfannotate, version 1.0.0-rc1.

### Variant filtering

**FIGURE 1:**
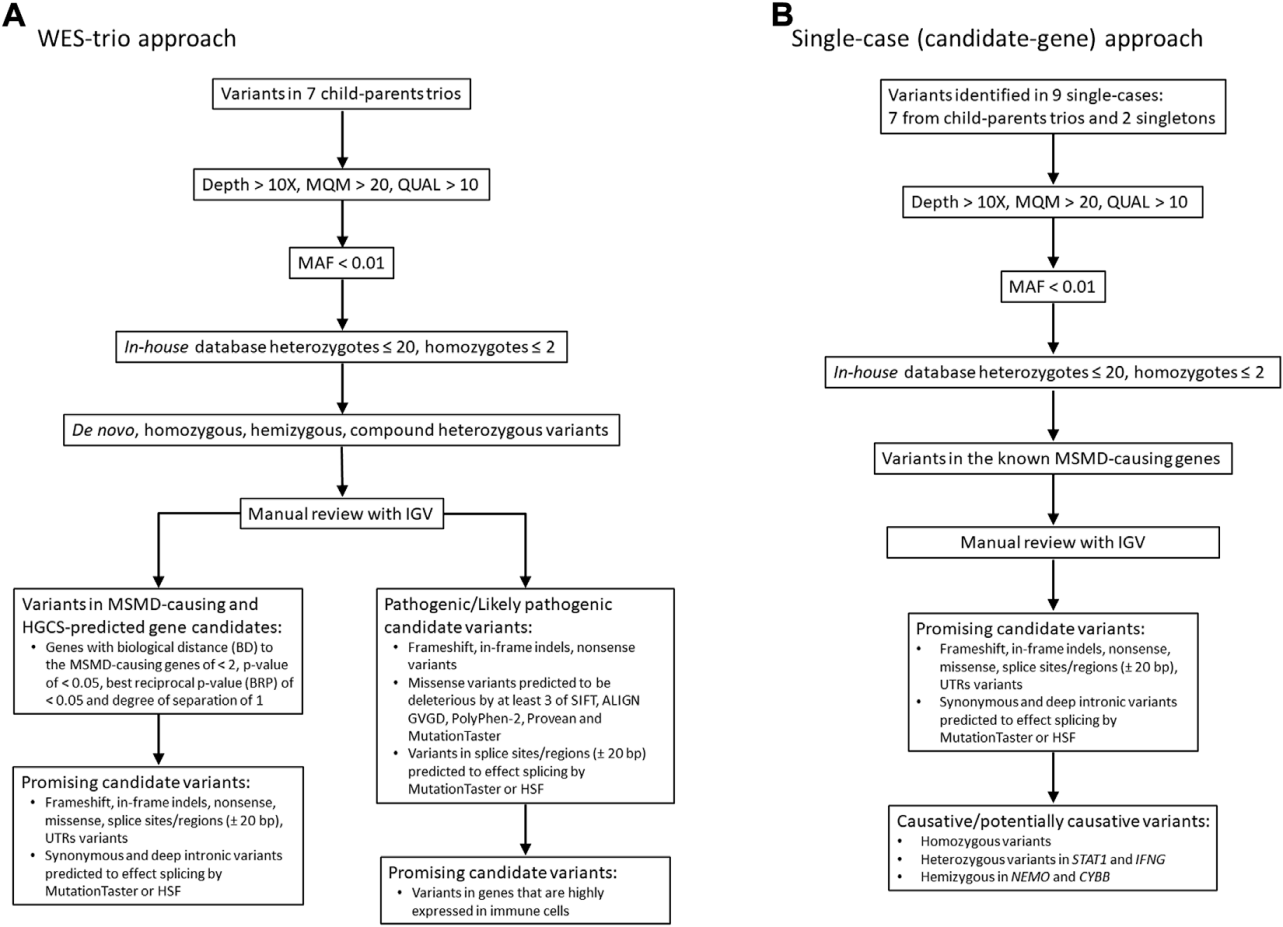
Workflow of WES analysis in 7 case-parental trios **(A)** and nine single cases **(B)**. The filtered candidate rare variants in single-case and trio-based approaches are shown in Table 2 and Table 3, respectively. See also supplementary material for filtering query results in each trio/single case (Supplementary Tables S2, S3). MQM = mean mapping quality; QUAL = variant quality.

The filtering of the resulting variants was performed using GSvar, Version 2018_03-12-gbe02570, following steps and criteria outlined in Figure 1. First, only variants with acceptable quality scores (MQM >20 and QUAL >10) were put through the filtering process. Variants were further annotated with population databases (1000G, ESP, ExAC, plus *in-house* database of variants, n = 900) and only rare variants (MAF cutoff of ≤0.01) with *in-house* occurrence ≤20 in heterozygous state and ≤2 in homozygous state were considered. We set up a MAF threshold of 0.01 (in the overall world population), because this value consistently explains the incidence of BCG-itis in the Republic of Moldova—15-20 cases per 30000-35000 newborns, assuming autosomal recessive inheritance of the disease. Of note, we primarily relied on the predicted pathogenicity of the variants rather than their frequencies, although we realize that the lower is the MAF the higher is the expected clinical effect.

Two filtering approaches were used for the analysis of WES data in this study (Figure 1). In seven of nine cases in which both parents were available (family cases), WES analysis was trio-based (an affected proband with unaffected parents). In this approach, the variants were filtered according to compound heterozygous, homozygous recessive, *de novo* dominant and X-linked inheritance models. All detected variants were then manually checked using the Integrative Genomics Viewer (IGV) to confirm their quality. Subsequently, genes were prioritized based on predicted pathogenicity of mutations and/or their biological proximity to the known MSMD-causing genes as described in next subsections.

In the single-case (or index-based) analysis, variants in 19 known MSMD-causing genes (*IL12B*, *IL12RB1*, *IL12RB2*, *ISG15*, *SPPL2A*, *IRF8*, *TYK2*, *FNGR1*, *IFNGR2*, *STAT1*, *KBKG*, *CYBB*, *JAK1*, *RORC*, *IL23R*, *ZNFX1*, *TBX21*, *IFNG* and *USP18*) were considered by using a filter for the MSMD gene panel. Additionally, a panel consisting of 26 pattern recognition receptor (PRR) genes (*TLR1*, *TlR2*, *TLR3*, *TLR4*, *TLR5*, *TLR6*, *TLR7*, *TLR8*, *TLR9*, *TLR10*, *CD14*, *NOD2*, *NLRP3*, *FCRLB*, *CLEC4E*, *CLEC7A*, *MRC1*, *CD209*, *MSR1*, *SCARB1*, *MARCO*, *CD36*, *STING1*, *AIM2*, *CGAS*, and *MBL2*), which are promising hereditary candidates affecting susceptibility to mycobacterial infections (Randhawa et al., 2011; Pöyhönen et al., 2015; Varzari et al., 2019; Dubé et al., 2021), was evaluated for the occurrence of rare variants. This index-case approach increases sensitivity, but with a loss of specificity. We therefore applied it to all cases (singletons and those from case-parents trios). All variants identified in MSMD and PRR gene panels were validated by visual inspection using IGV.

### In-silico prediction

Detailed *in silico* predictions for identified coding non-synonymous (missense) mutations were made with tools Align GVGD (Tavtigian et al., 2008), SIFT (Sim et al., 2012), MutationTaster (Schwarz et al., 2010), Provean (Choi and Chan, 2015) and Polyphen2 (Adzhubei et al., 2010) implemented in ALAMUT visual (interactive biosoftware, Version 2.7 rev.1, Rouen, France). The missense variants which were predicted as deleterious by at least three out of five methods were assumed likely as pathogenic. For predicting splicing effects, MutationTaster and the Human Splicing Finder (HSF) software (http://www.umd.be/HSF3/) were used (Desmet et al., 2009), and the overall effect was considered deleterious if at least one tool predicts it to be deleterious. Information from the variant genetics database ClinVar (https://www.ncbi.nlm.nih.gov/clinvar/), which adhere to the guidance of the American College of Medical Genetics (ACMG), was also employed for all types of variants.

The effects of mutations on protein structure were assessed using ExPASy translate tool (https://www.swissmodel.expasy.org/) (Waterhouse et al., 2018). In addition, I-Mutant 2.0 software (http://folding.biofold.org/i-mutant/i-mutant2.0.html) was used to predict the impact of mutations on protein structure stability based on the change in Gibbs free energy (ΔΔG) (Capriotti et al., 2006); the predictions were performed starting from the protein 3D structure with the temperature of 36°C and pH of 7.0 as default parameters. Finally, the Oligogenic Resource for Variant AnaLysis (ORVAL) platform was used to predict clinical consequences of intergenic interactions (https://orval.ibsquare.be/). Both heterozygous and homozygous rare variants identified through candidate genes and trio-based approaches were analyzed in ORVAL in accordance with recommendations of the developers (Renaux et al., 2019).

### Functional annotation and categorization of genes

We used DAVID classification system (https://david.ncifcrf.gov/) and PubMed (https://pubmed.ncbi.nlm.nih.gov/) for information about gene function and their involvement in diseases.

Biological affinity/similarity between candidate genes identified by the trio-based prioritization analysis and 19 known MSMD-causing genes was evaluated using the Human Gene Connectome Server (HGCS) (https://hgc.rockefeller.edu/) (Itan et al., 2014). The level of protein-protein/gene-gene interaction was also analyzed and graphically visualized as nodes (genes/proteins) and edges (the relationship between proteins) using STRING software (https://string-db.org/). In this analysis, the novel and known MSMD-causing genes were considered as the seed molecules from which direct and indirect protein-protein/gene-gene interactions were obtained in accordance with the developers’ recommendations (Szklarczyk et al., 2019).

The genes were also categorized based on their expression pattern as reported in the GTEx database (GenotypeTissue Expression Project database, https://gtexportal.org/home/). Genes with the higher expression levels in whole blood or immune-related cells (i.e., spleen or EBV-transformed lymphocytes) were given the highest priority in this study. Additionally, RD-Match (http://genetics.bwh.harvard.edu/rdmatch/) was used to estimate probability of finding mutation by chance in a given gene (Akle et al., 2015).

### Whole blood RNA extraction and RT-PCR

Peripheral blood samples were collected *via* PAXgene RNA tubes (Qiagen, Valencia, CA) and stored at - 80°C until RNA extraction. RNA was extracted *via* PreAnalytiX PAXgene blood RNA kit from PreAnalytiX/Qiagen. DNase I treatment was performed at the same time as the RNA isolation using the reagents from the PreAnalytiX PAXgene blood RNA kit. Quantity and quality of the extracted RNA was verified by the NanoDrop^®^ ND-1000 spectrophotometer (NanoDrop Technologies, Wilmington, DE. United States) and the Agilent 2100 Bioanalyzer (Agilent Technologies, Santa Clara, CA. United States).

RNA was reverse transcribed into cDNA using SuperScript^®^ VILO Master mix (Invitrogen, Massachusetts, United States). Polymerase chain reactions (PCR) of cDNA sequences flanking variants *IL12B* c.877A>G (rs1177184657), IL12B c.89–14T>C, *HEATR3* c.395_396delAA, *STAT1* c.373–2A>C and *GBP2* c.1149+14T>C were performed using Tempase Hot Start DNA Polymerase (Ampliqon, Odense, DK) and specific primers: 5′-AGA​GCA​AGA​TGT​GTC​ACC​AG-3′ and 5′-TGA​TTG​TCG​TCA​GCC​ACC​AG-3′ for *IL12B* c.89–14T>C, 5′-CAT​CAG​GGA​CAT​CAT​CAA​ACC-3′ and 5′-TCC​ATA​CAT​CCT​GGC​AGA​CA-3′ for *IL12B* c.877A>G, 5′-TCT​GGA​AAA​CGC​CCA​GAG​ATT-3′ and 5′-ATT​GGT​CTC​GTG​TTC​TCT​GTT​CT-3′ for STAT1 c.373–2A>C, 5′-TGC​TGG​AAA​AGC​TCC​AGC​ACC-3′ and 5′-TCC​ACA​GCA​CGT​TCA​CAG​TC-3′ for *HEATR3* c.395_396delAA, and 5′-AGG​AGG​AAG​AGC​TGA​ACC​CT-3′ and 5′-GTC​ATC​TCG​CCT​TGC​TTC​C-3′ for *GBP2* c.1149+14T>C. The PCR products were separated and verified by electrophoresis on agarose gel. In case alternative splice variants were identified, the bands were purified by gel extraction using Qiagen Gel Extraction kit (Qiagen, Hilden, DE) and sequenced using the dideoxy chain-terminator method of Sanger.

### GBP2 minigene reporter assay

A minigene splicing assay was performed to verify/validate whether the candidate mutation c.1149+14T>C in *GBP2* affected splicing products. Briefly, a 1,157 bp fragment harboring exon 7 (281 bp) and part of the flanking introns (246 bp of intron 6 and 581 bp of intron 7) was amplified by standard PCR with patient genomic DNA as template employing a proofreading polymerase and primers: GBP2S-NotI (5′-AAT​CGG​GCG​GCC​GCT​TGA​CCT​AGT​ATC​TCA​GCC​CCA) and GBP2AS-BamH (5′-CGA​TTC​GGA​TCC​CCA​GAA​CCT​CTG​ACT​GTA​GCA). The fragment was cloned into the pSPL3 reporter vector between the NotI and BamHI sites (Life Technologies).

Sanger sequencing was used to evaluate whether the wild-type (WT) and mutant-type (MT) expression vectors had been successfully constructed without errors. The minigenes were transfected in duplicates into HepG2 cells using X-tremeGENE transfection reagent (Roche Life Science) according to the manufacturer’s instructions and our previous publications (Palhais et al., 2015; Palhais et al., 2016). Forty 8 hours after transfection cells were harvested and RNA was isolated with QIAzol (Qiagen) and chloroform extraction. cDNA synthesis was performed using SuperScript^®^ VILO Master mix (Invitrogen). Splicing of *GBP2* exon seven was examined by PCR using Tempase Hot Start DNA Polymerase (Ampliqon) and vector specific primers SD6 (5′-TCT​GAG​TCA​CCT​GGA​CAA​CC-3′) and SA2 (5′-ATC​TCA​GTG​GTA​TTT​GTG​AGC-3′). The PCR products were separated and verified by electrophoresis in an agarose gel and characterized by direct Sanger sequencing. The relative quantity of the bands corresponding to exon inclusion/exon skipping was estimated by capillary electrophoresis using the Fragment Analyzer™ (Agilent Technologies, Santa Clara, United States) instrument and software and reported as percent exon skipping (relative to the total intensity of the signal in each lane).

### Real-time qPCR

The expression of *GBP2* was analyzed by quantitative real time PCR in five RNA samples: two independently isolated RNA samples from the carrier of the mutation c.1149+14T>C (i.e., the father of the patient P2) and three matched control samples. All reactions were run in duplicate on the Roche LightCycler 480 with SYBR-green premix FastStart Essential DNA Green Master (Roche Diagnostics, CH) according to manufacturer instructions employing the following primer pairs: GBP2 forward 5′-CAA​TTA​CGC​AGC​CTG​TGG​TG-3′, GBP2 reverse 5′- GAG​CCT​AGA​GAG​AAG​CCG​TTT and RPL13A forward 5′- AAG​GTC​GTG​CGT​CTG​AAG, RPL13A reverse 5′-GAG​TCC​GTG​GGT​CTT​GAG. *RPL13A* was used as a normalization control because it is stably expressed across different tissue types, including blood cells (Ledderose et al., 2011; Sabatino et al., 2013; Xiao et al., 2017; Dembic et al., 2019).

### Statistical analysis

Data from RT-qPCR are expressed as mean ± standard error (s.e.m). Samples were normalized to RPL13A. Means of different groups were compared and analyzed using the Student’s t-test.

### Surface plasmon resonance imaging (SPRi)

SPRi was carried out as previously described (Holm et al., 2022). Briefly, biotinylated RNA-oligonucleotides (GBP2-WT: 5′-UAUUUGGUGUUCUACGUCAUGA-teg-bio and GBP2-MUT: 5′-UAUUUGGUGUCCUACGUCAUGA-teg-bio) were immobilized onto a G-strep sensorchip (SSENS) for 20 min. The hnRNPA1 recombinant protein were injected for 8 min, followed by dissociation for 4 min; 6.25–200 nM hnRNPA1 (Abcam, ab123212). A continuous flow of SPR buffer (10 mM Tris-HCl, ph 7.9, 150 mM KCl, 3.4 mM EDTA, 0.005% tween-20). Binding was fitted to a 1:1 kinetics model with Scrubber2 (v. 2.1.1.0; Biologics inc.). For hnRNPA1 a biphasic 1:2 model was used in ClampXP (version 3.50; Biosensor Data Analysis).

## Results

### Summary of exome sequencing data

Overall, 100 M raw reads per sample (range 76M–229 M) were generated, of which 98.7% (range 97.8%–99.5%) and 81.1% (range 74.11%–85.93%) were aligned to the human reference genome (hg19) with a mapping qualities of Q20 and Q30, respectively (Supplementary Table S1). On average, 97.9% of the targeted regions (range 95.3%–98.8%) were covered at least 10 times, and 97.0% of the targeted regions (range 90.6%–98.5%) were covered at least 20 times. The average read depth in the targeted region was 129x (range 96.7x–244.9x) reads per base. Above all, these data fully reflect the reliability of our sequencing, thus providing a solid basis for the follow-up analysis.

Two approaches (trio-based exonic screening and single cases candidate genes analysis) were employed to identify disease-associated variants (Figure 1). The filtering query results for each sample are given in Supplementary Tables S2, S3.

### Variants in MSMD and PRR gene panels

Twelve qualifying low frequency variants within eight known MSMD-causing genes were identified in six of the nine patients investigated, including six missense, three splice site/region (±20 bp), two deep intronic and one synonymous mutations (Table 2 and Supplementary Table S3). All detected mutations were in a single heterozygous state. Three of them were found in genes *STAT1* (c.373-2A>C in P9 and c.541+50T>C in P7) and *IFNG* (c.40G>A/p.(Val14Met) in P3), responsible for both autosomal dominant and autosomal recessive forms of mycobacterial disease. Three variants were found in the *IL12B* gene in two patients, with variants c.877A>G/p.(Lys293Glu) and c.89-14T>C present in the same patient (P6) in a compound heterozygous state (according to the trio-based analysis). Noteworthy, five variants, namely *TYK2* c.2441C>T, *TYK2* c.157G>A, *IFNGR1* c.40G>A, *IL23R* c.257G>A, *IL12B* c.961G>A and *IL12RB1* c.16196C>T, have been previously reported in patients with MSMD, but it is unclear whether they are actually connected to the disease (https://www.ncbi.nlm.nih.gov/clinvar/).

**TABLE 2:** Rare variants (MAF <0.01) identified in the known MSMD-associated genes.

Patient	Gene	Nucleotide change (transcript GRCh37/hg19)^a^	Amino acid change	Type of mutation	rs ID	GnomAD	ALIGN GVGD (class)^b^	SIFT	Mutation taster	PolyPhen-2	Provean	HSF splice prediction	ClinVar interpritationn (number of submitters)	OMIM	Reported inheritance
**P2**	*TYK2*	NM_003331.5:c.2441C>T	p.(Pro814Leu)	Missense	rs143743593	0.0001255	C65	D	DC	PrD	D	No significant impact on splicing signals	VUS (2)	176941	AR
	*ZNFX1*	NC_000020.10:g.47877143G>A (NM_021035.3:c.2302–29C>T)	p.(=)	Intron	rs369214366	0.0004319	-	-	P	-	-	No significant impact on splicing signals	-	619644	AR
**P3**	*IFNGR1*	NM_000416.3:c.40G>A	p.(Val14Met)	Missense	rs11575936	0.001253	C0	T	P	PrD	N	Activation of a cryptic Donor site. Potential alteration of splicing	VUS (1)/B (1)	107470	AR/AD
	*IL23R*	NM_144701.3:c.257G>A	p.(Arg86Gln)	Missense	rs76575803	0.003306	C0	T	P	B	N	Activation of a cryptic Acceptor site. Potential alteration of splicing	B (1)	607562	AR
	*JAK1*	NM_002227.4:c.414C>T	p.(=)	Synonimous	rs35237903	0.001699			P			No significant impact on splicing signals	LB (1)	147795	AR
**P6**	*IL12B*	NM_002187.3:c.877A>G	p.(Lys293Glu)	Missense	rs1177184657	0.000004037	C0	T	P	B	N	Significant alteration of ESE/ESS motifs ratio (9)	-	161561	AR
		NC_000005.9:g.158750351A>G (NM_002187.3:c.89–14T>C)	p.(=)	Intron and splice region	NA	0	-	-	P	-	-	Significant alteration of ESE/ESS motifs ratio (7)	-		
	*TYK2*	NM_003331.5:c.157G>A	p.(Ala53Thr)	Missense	rs55762744	0.007301	C0	D	DC	PrD	D	No significant impact on splicing signals	B (3)	176941	AR
**P7**	*STAT1*	NC_000002.11:g.191864302A>G (NM_007315.4:c.541+50T>C)	p.(=)	Intron	rs41379748	0.008948	-	-	P	-	-	No significant impact on splicing signals	-	600555	AR/AD
**P8**	*IL12B*	NM_002187.3:c.961G>A	p.(Glu321Lys)	Missense	rs201512006	0.00008903	C0	T	P	B	N	No significant impact on splicing signals	VUS (2)	161561	AR
**P9**	*STAT1*	NC_000002.11:g.191865891T>G (NM_007315.4:c.373–2A>C)	p.(=)	Splice acceptor	NA	0	-	-	DC	-	-	Alteration of the WT Acceptor site, most probably affecting splicing	-	600555	AR/AD
	*IL12RB1*	NC_000019.9:g.18173093G>A (NM_005535.3:c.1619–6C>T)	p.(=)	Intron and splice region	rs530116677	0.00007604	-	-	P	-	-	No significant impact on splicing signals	LB (1)	601604	AR

D = deleterious; DC, disease causing; PrD = probably damaging; T = tolerated; P = polymorphism; B = benign; LB = Likely benign; N = neutral; VUS, variant of uncertain significance; AR, autosomal recessive; AD, autosomal dominant.

^a^ Variant nomenclature followed HGVS recommendations.

^b^ For ALIGN GVGD, Class C0 indicates that a change is unlikely to be pathogenic, whereas class C65 represents the highest likelihood of a change to be pathogenic.

The study also identified twelve variants in nine PRR genes in six patients (Supplementary Table S4). All were single heterozygous changes, inherited (as seen from the case-parents trio analysis), reported in databases with little or no functional consequences (i.e., intronic, synonymous, benign missense variants).

### Trio-based exonic screening of rare variants and phenotype-based gene prioritization

Using trio-based WES, a total of 455 high-confidence rare variants in 201 genes that fit the proposed inheritance criteria (i.e., *de novo*, homozygous, hemizygous, compound heterozygous variants) were detected in seven trios. These variants included 389 compound heterozygous mutations in 135 genes, 20 homozygous (6 missense, four splice region intronic, one UTR, three intronic, five synonymous, one in non-coding transcript), 32 X-linked hemizygous (12 missense, one in-frame insertion, four splice region intronic, seven UTRs, two intronic, six synonymous), and 14 *de novo* (4 missense, one frame shift, one in-frame deletion, one splice region, two intronic, five synonymous) variants. After *in-silico* assessment of pathogenicity, 20 most promising variants in 13 genes were finally selected as top candidates for susceptibility to mycobacterial disease (Tables 3 and 4; Supplementary Table S5).

**TABLE 3:** Candidate rare variants (MAF <0.01) identified in the seven MD trio probands.

Patient/trio	Gene	Nucleotide change (transcript GRCh37/hg19)^a^	Protein variant	Type of mutation	rs ID	Zygocity	Inherited from	GnomAD	ALIGN GVGD (class)^b^	SIFT	Mutation taster	PolyPhen-2	Provean	HSF splice prediction	ClinVar interpritationn (number of submitters)	Link to the MSMD-causing genes^c^
**P1**	*LRP1B*	NM_018557.3:c.11227G>A	p.(Gly3743Ser)	Missense	rs150879175	Com-het	Mother	0.006697	C0	T	DC	PrD	D	Activation of a cryptic Donor site. Potential alteration of splicing	B (1)	-
		NM_018557.3:c.1907G>A	p.(Arg636Gln)	Missense	rs77234491	Com-het	Father	0.001996	C0	T	DC	PrD	D	Activation of a cryptic Acceptor site. Potential alteration of splicing	LB (1)	
	*KDM6A*	NM_021140.4:c.1402T>C	p.(Cys468Arg)	Missense	rs138723332	Hemi	Mother	0.0005388	C0	D	DC	B	D	No significant impact on splicing signals	B (1)	-
	*H2BW2*	NM_001164416.3:c.368T>G	p.(Leu123Arg)	Missense	rs782603545	Hemi	Mother	0.000129	C0	D	DC	PrD	D	Significant alteration of ESE/ESS motifs ratio (-4)	-	-
**P2**	*GBP2*	NC_000001.10:g.89579685A>G (NM_004120.5:c.1149+14T>C)	p.(=)	Intron and splice region	rs753229736	Com-het	Father	0.0001458	-	-	P	-	-	No significant impact on splicing signals	-	*IRF8*, *ISG15*, *STAT1*
		NM_004120.5:c.412G>A	p.(Ala138Thr)	Missense	rs138509928	Com-het	Mother	0.001998	C0	D	DC	PrD	D	No significant impact on splicing signals	-	
	*RNF123*	NC_000003.11:g.49753164C>T (NM_022064.5:c.3150+17C>T)	p.(=)	Intron and splice region	NA	Het	*De novo*	0	-	-	P	-	-	Activation of a cryptic Donor site. Potential alteration of splicing	-	-
**P3**	*PPP1R9B*	NC_000017.10:g.48227115_48227123del (NM_032595.5:c.759_767del)	p.(Pro252_Pro254del)	Disruptive in frame deletion	rs1486078473	Het	*De novo*	0	-	-	-	-	-	-	-	-
**P4**	*TTN*	NM_133378.4:c.77167C>T	p.(Arg25723Cys)	Missense	rs192152102	Com-het	Father	0.0007143	C0	D	DC	PrD	D	No significant impact on splicing signals	VUS (6)/LB (2)/B (5)	-
		NM_133378.4:c.68129G>A	p.(Arg22710His)	Missense	rs769729114	Com-het	Mother	0.00004659	C0	D	DC	PrD	D	Significant alteration of ESE/ESS motifs ratio (3)	VUS (2)	
**P5**	*ZCWPW1*	NM_017984.6:c.1049A>G	p.(His350Arg)	Missense	rs145418256	Com-het	Mother	0.002298	C0	D	DC	PsD	D	No significant impact on splicing signals	-	-
		NM_017984.6:c.314A>G	p.(Glu105Gly)	Missense	rs141450215	Com-het	Father	0.005738	C0	D	P	PrD	D	No significant impact on splicing signals	-	
	*SRPX2*	NM_014467.3:c.517G>A	p.(Glu173Lys)	Missense	rs764018303	Hemi	Mother	0.00002213	C15	D	DC	PsD	N	No significant impact on splicing signals	-	-
	*RBMXL3*	NM_001145346.2:c.128G>A	p.(Arg43Gln)	Missense	rs201501183	Hemi	Mother	0.001478	C0	D	P	PrD	D	Activation of a cryptic Donor site. Potential alteration of splicing	-	-
**P6**	*IL12B*	NM_002187.3:c.877A>G	p.(Lys293Glu)	Missense	rs1177184657	Com-het	Mother	0.000004037	C0	T	P	B	N	Significant alteration of ESE/ESS motifs ratio (9)	-	MSMD-causing gene
		NC_000005.9:g.158750351A>G (NM_002187.3:c.89–14T>C)	-	Intron and splice region	NA	Com-het	Father	0	-	-	P	-	-	Significant alteration of ESE/ESS motifs ratio (7)	-	
	*HEATR3*	NM_182922.4:c.395_396delAA	p.(Lys132Argfs*3)	Frame shift	NA	Het	*De novo*	0	-	-	-	-	-	1. Significant alteration of ESE/ESS motifs ratio (-6) 2. Activation of a cryptic Donor site. Potential alteration of splicing Alteration of the WT Donor site, most probably affecting splicing	-	-
	*IFNW1*	NM_002177.3:c.58G>A	p.(Gly20Arg)	Missense	rs34158787	*Homo*	Both parents	0.003384	C0	T	P	B	D	NA	-	*IFNGR1, IFNGR2, IL12RB1, IL12RB2, IL23R*
**P7**	*TTN*	NM_133378.4:c.97070A>C	p.(Glu32357Ala)	Missense	rs201218828	Com-het	Mother	0.001661	C0	D	DC	PrD	D	Significant alteration of ESE/ESS motifs ratio (3)	VUS (4)/LB (8)/B (4)	-
		NM_133378.4:c.75185A>C	p.(Gln25062Pro)	Missense	rs375607506	Com-het	Father	0.00001788	C0	D	DC	B	D	Significant alteration of ESE/ESS motifs ratio (4)	NA	
	*GAL3ST2*	NC_000002.11:g.242716400G>A (NM_022134.3:c.29+1G>A)	p.(=)	Splice donor	rs78620448	Com-het	Mother	0.003680	-	-	DC	-	-	Alteration of the WT Donor site, most probably affecting splicing	VUS (1)/LB (1)	-
		NM_022134.3:c.458A>G	p.(Tyr153Cys)	Missense	rs139344622	Com-het	Father	0.004521	C0	D	P	PrD	D	Significant alteration of ESE/ESS motifs ratio (-4)	-	
	*HMCN1*	NM_031935.3:c.109G>A	p.(Glu37Lys)	Missense	rs199574143	Com-het	Father	0.0001626	C0	D	DC	PrD	N	No significant impact on splicing signals	-	-
		NM_031935.3:c.4586A>G	p.(Asn1529Ser)	Missense	rs41317471	Com-het	Mother	0.005867	C0	D	DC	PrD	N	Activation of a cryptic Acceptor site. Potential alteration of splicing	LB (1)/B (2)	
	*SIGLEC6*	NM_001245.7:c.292G>T	p.(Asp98Tyr)	Missense	rs62617068	*Homo*	Both parents	0.008596	C0	D	P	PrD	D	Activation of a cryptic Donor site. Potential alteration of splicing	B (1)	-

Com-het = compound heterozygous; Het = heterozygous; *Homo* = homozygous; Hemi = X-linked hemizygous; D = deleterious; DC, disease causing; PrD = probably damaging; PsD = possibly damaging; T = tolerated; P = polymorphism; B = benign; LB, likely benign; N = neutral; VUS, variant of uncertain significance.

^a^ Variant nomenclature followed HGVS recommendations.

^b^ For ALIGN GVGD, Class C0 indicates that a change is unlikely to be pathogenic, whereas class C65 represents the highest likelihood of a change to be pathogenic.

^c^ Genes are predicted by HGCS., Core genes used in prediction: *IL12RB1*, *IL12B, IL12RB2, ISG15*, *SPPL2A*, *IRF8*, *TYK2*, *FNGR1*, *IFNGR2*, *STAT1*, *IKBKG*, *CYBB*, *JAK1*, *RORC*, *IL23R, ZNFX1, TBX21, IFNG*, and *USP18*; only genes with biological distance (BD) to the core genes of <2, *p*-value of <0.05, best reciprocal *p*-value (BRP) of <0.05 and degree of separation of one are considered.

**TABLE 4:** Summary of the novel genes with rare variants detected in the trio-based WES analysis.

Gene	OMIM/HGNC	Protein	Molecular function(s)^a^	Biological process (es)^a^	Associated disease(s)^b^	Reported link to infectious diseases^b^	Reported link to the immune system disorders^b^
*TTN*	188840/12403	Titin	Serine/threonine-protein kinase activity, cytoskeleton protein, cytoskeletal proteins binding	Sarcomere organization, striated muscle contraction; mitotic chromosome condensation and segregation	Hypertrophic cardiomyopathy; dilated cardiomyopathy; limb-girdle muscular dystrophy, distal myopathy; tibial muscular dystrophy	No	No
*GAL3ST2*	608237/24869	Galactose-3-O-sulfotransferase 2	Sulfotransferase and galactosylceramide sulfotransferase activities	Glycoprotein and glycolipid biosynthetic process	Implicated in tumor metastasis processes	No	No
*HMCN1*	608548/19194	Hemicentin-1	Extracellular matrix glycoprotein, calcium ion binding	Cell cycle; cell division; sensory transduction	Age-related macular degeneration 1	No	No
*GBP2*	4183/600412	Guanylate binding protein 2	GTP binding and GTPase activity	IFN-γ and type I interferon signaling; defense response to bacterium	Several types of cancer; several infectious diseases, including mycobacteriosis	Yes	Yes
*ZCWPW1*	23486/618900	Zinc finger CW-type and PWWP domain containing 1	Methylated histone binding	Cell differentiation; spermatogenesis	Late-onset Alzheimer disease	No	No
*LRP1B*	6693/608766	LDL receptor related protein 1B	Calcium ion binding, low-density lipoprotein receptor activity	Receptor-mediated endocytosis; lipoprotein transport	Several types of cancer; SLE	No	Yes
*PPP1R9B*	603325/9298	Neurabin 2, spinophilin	Actin filament binding; protein kinase activity; protein phosphatase inhibitor activity	A scaffold protein in multiple signaling pathways, including cellular differentiation and neurogenesis	Progression and malignancy of different tumors; chronic rhinosinusitis	No	Yes
*HEATR3*	614951/26087	HEAT repeat containing 3	Unfolded protein binding	Protein import into nucleus; ribosomal large subunit biogenesis and assembly; NOD2-mediated NF-kappaB signaling	Crohn’s disease; esophageal cancer; uterine fibroids	No	Yes
*SRPX2*	300642/30668	Sushi Repeat Containing Protein X-Linked 2	Hepatocyte growth factor binding; signaling receptor binding	Cellular migration and adhesion; angiogenesis; synapse assembly	Rolandic epilepsy; mental retardation; speech dyspraxia; pulmonary fibrosis; several types of cancer; abdominal aortic aneurysms	No	No
*RBMXL3*	NA/26859	RBMX like 3	RNA binding	mRNA splicing	Colorectal liver metastases	No	No
*KDM6A*	300128/12637	Lysine demethylase 6A	Chromatin binding; oxidoreductase activity; metal ion binding	Chromatin remodelling; histone demethylation/methylation; regulation of gene expression	Kabuki syndrome; several types of cancer; obesity and metabolic syndrome; viral infections	Yes	Yes
*H2BW2 (H2BFM)*	NA/27867	H2B.W histone 2	DNA binding	Nucleosome assembly	No	No	No
*SIGLEC6*	604405/10875	Sialic acid binding Ig like lectin 6	Carbohydrate binding; sialic acid binding	Cell adhesion; cell-cell signaling, including immunoregulatory interactions	Preeclampsia; gestational trophoblastic disease; leukemia; SLE	No	Yes
*IFNW1*	147553/5448	Interferon omega-1	Type I interferon receptor binding; cytokine activity	Type I interferon signaling; defense response to virus	Several types of cancer; viral infections; autoimmune polyglandular syndrome type 1	Yes	Yes
*RNF123*	614472/21148	Ring finger protein 123	Ubiquitin-protein transferase activity	Metabolism of protein; antiviral signaling	Glioblastoma; depressive disorder; chronic widespread musculoskeletal pain; lymphoma; melanoma	No	Yes

NA, not available.

^a^ UniProtKB/Swiss-Prot Summary.

^b^ Keyword (gene-name) -based search in the NCBI/PubMed database.

^c^ Genes are predicted by HGCS., Core genes used in prediction: *IL12RB1, IL12B, IL12RB2, ISG15, SPPL2A, IRF8, TYK2, FNGR1, IFNGR2, STAT1, IKBKG, CYBB, JAK1, RORC*, *IL23R, ZNFX1, TBX21, IFNG*, and *USP18*; only genes with biological distance (BD) to the core genes of <2, *p*-value of <0.05, best reciprocal *p*-value (BRP) of <0.05 and degree of separation of one are considered.

**FIGURE 2:**
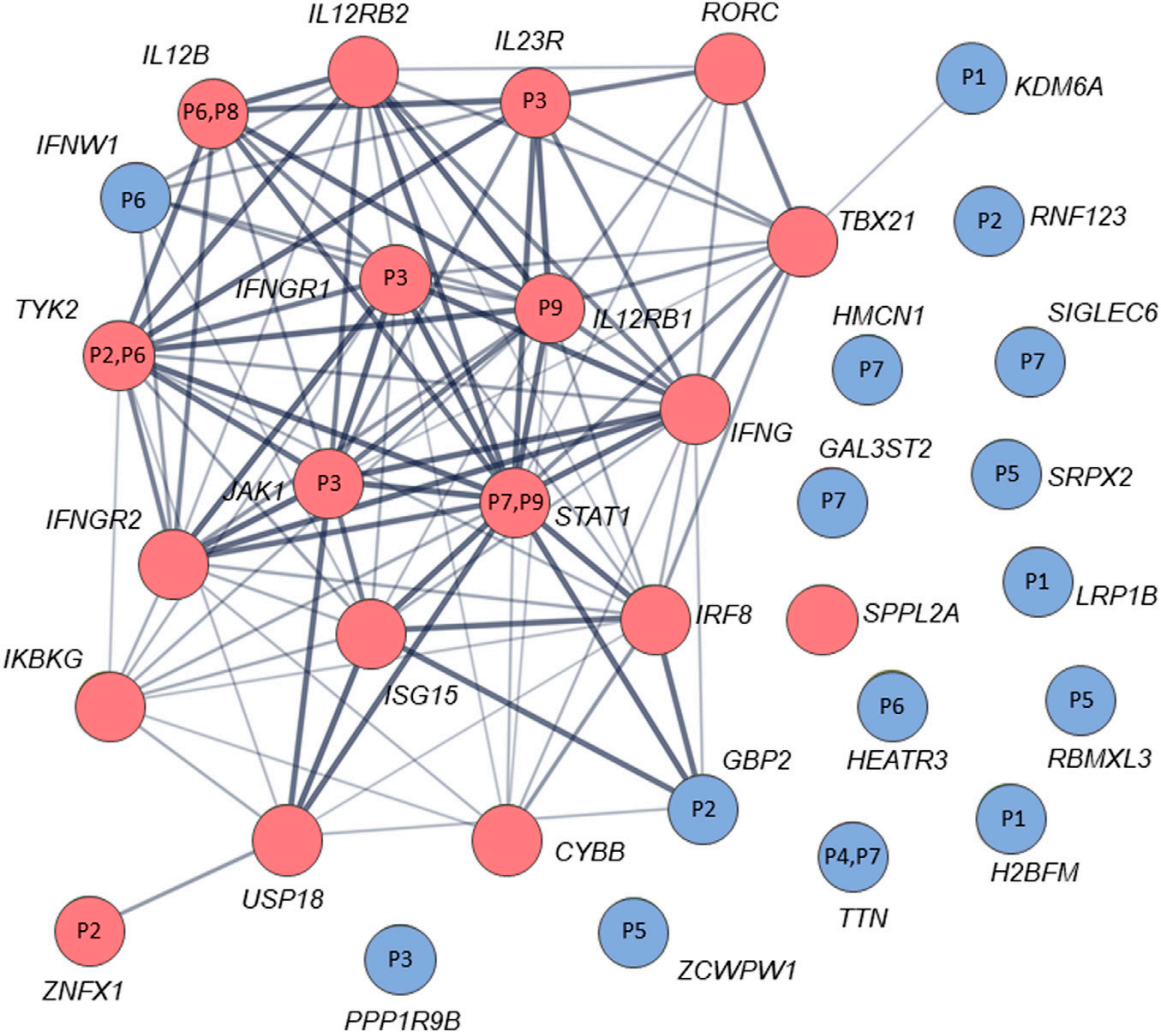
STRING interaction (network) analysis (https://string-db.org/) between 34 genes/proteins. Each circle represents a protein/gene: red nodes are known MSMD-causing genes and the blue nodes are novel candidate genes identified in this study *via* trio-based approach. Numbers inside the nodes denote codes for patients with mutations in the corresponding genes. Interactions between proteins/genes are indicated by lines with variable thickness showing the strength of data support. The confidence cutoff for showing interaction links has been set to ‘medium’ (0.400).

By analysis of biological distances between the identified 201 genes and MSMD core genes using HGCS software, two genes–*GBP2* and *IFNW1* were ranked as the closest to the MSMD genes. Two heterozygous variants in *GBP2* (c.412G>A/p.(Ala138Thr) and c.1149+14T>C) and one homozygous variant in *IFNW1* (c.58G>A/p.(Gly20Arg)) were therefore prioritized (Table 3). Similar results were obtained by analysis of gene network using String software (Figure 2). In this analysis, genes *GBP2* and *IFNW1* are closely interconnected with other known MSMD-associated genes, forming a densely connected module, which implies their direct functional involvement in the disease-associated (i.e., IFN-γ signaling) pathway. Also, *KDM6A* showed some affinity with the MSMD-associated gene *TBX21*. Other genes found in this study locate outside the network, suggesting either their affiliation to different pathways or indirect interactions *via* yet unknown elements.

The significance of new candidate genes was also assessed by their expression in immune related tissues using the GTEx database. Of 15 prioritized in this study genes, four genes, namely *GBP2*, *HEATR3*, *PPP1R9B* and *KDM6A*, exhibited an elevated transcriptional level in whole blood, spleen or EBV-transformed lymphocytes (Supplementary Table S6), thus lending additional support to their role in increasing susceptibility to mycobacterial infections. Remarkably, genes *HEATR3* and *PPP1R9B* harbor *de novo* variants, both with predicted damaging effects–a frameshift (Lys132Argfs*3) and a disruptive in frame deletion (Pro252_Pro254del), respectively. Assessment with RD-Match did not reveal true-positive matches between immunodeficiency and either our or known MSMD-associated genes (Supplementary Table S7), thus indicating its insufficient power for mitigating false-positive findings in this study.

### Analysis of mRNA transcripts

Two patients were available for the analysis of mRNA, including one with BCG-tis (P6) and another with disseminated TB infection (P9). For the four candidate mutations identified in a patient with BCGitis, effects on splicing were predicted for two compound heterozygous variants c.89-14T>C and c.877A>G in the known MSMD-associated gene *IL12B* and one *de-novo* frameshift mutation c.395_396delAA in *HEATR3*. To examine the functional impact of these variants on mRNA splicing, we performed RT-PCR experiments on total RNA extracted from peripheral leukocytes. Agarose gel electrophoresis showed only single wild-type bands across four loci in all samples (the carriers and controls), indicating no visible effect of these mutations on the pre-mRNA splicing (Supplementary Figure S1).

**FIGURE 3:**
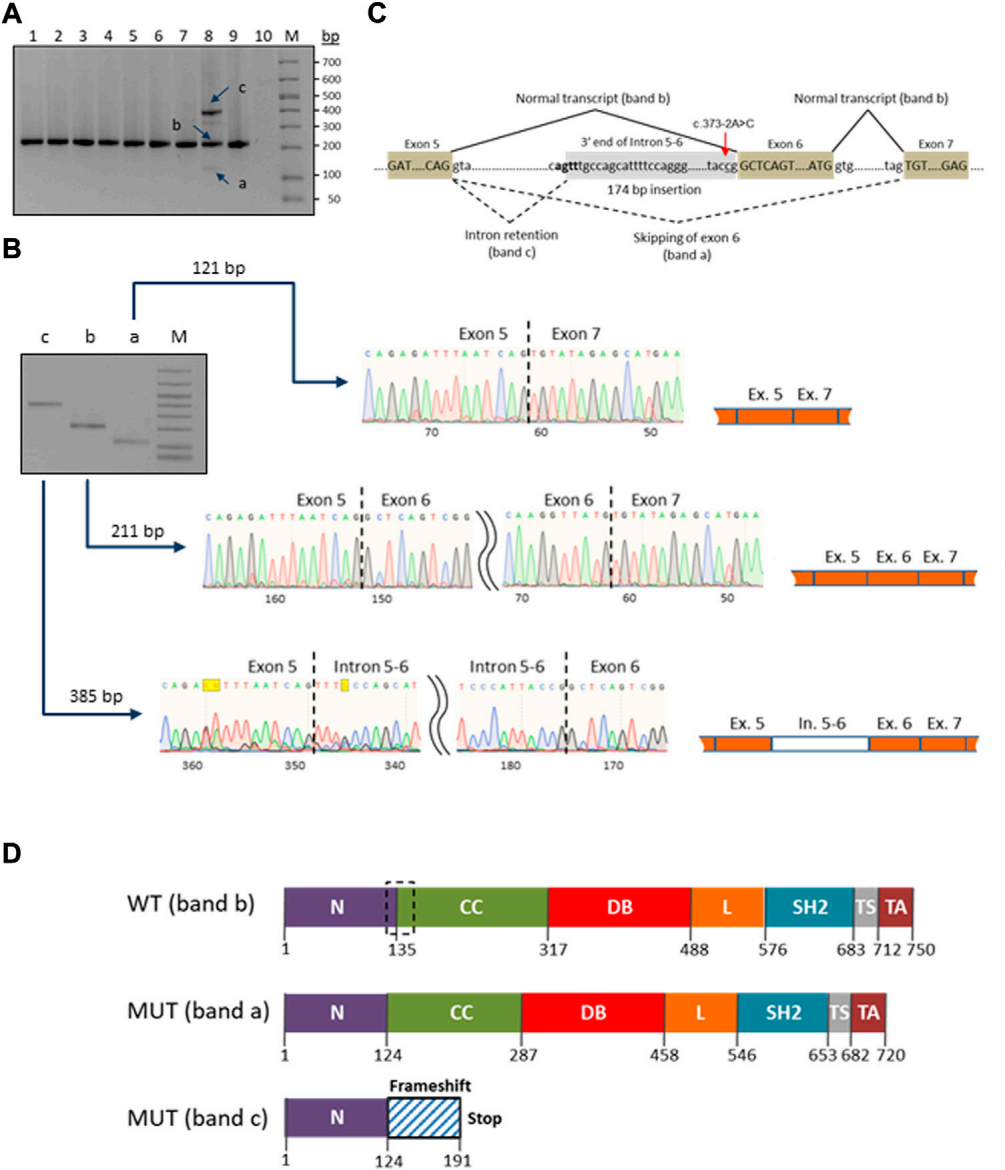
Characterization of the novel c.373–2A>C mutation in the *STAT1* gene. **(A)** RT-PCR electrophoresis products on 2% agarose gel. The carrier patient (lane 8) showed three cDNA fragments (a, b and c) compared to one in the controls (lanes 1-7 and 9). **(B)** Purification and Sanger sequencing of RT-PCR products. Sanger sequencing revealed correctly spliced STAT1 transcript of 211 bp (lane b) and two mutated transcripts: an aberrant short fragment (121 bp, lane a) corresponding to the in-frame skipping of exon six and the aberrant long fragment (385 bp, lane c) corresponding to the out-of-frame retention of the intronic fragment. **(C)** Schematic representation of the splicing for the splice site mutation c.373-2A>C (red arrow). The newly generated splice signal between exons 5-6 is shown in bold. Exons are shown as boxes. **(D)** Prediction of impact of the splice site mutation c.373-2A>C on the STAT1 protein structure. Wild-type STAT1 is a multidomain protein of 750 amino acids. The mutant sequences are of 720 and 191 ammino acids length, corresponding to the short (exon six skipping/band a) and long (174 bp intronic fragment insertion/band c) transcripts/bands, respectively. The structural domains within the protein are the coiled-coil domain (CC), DNA binding domain (DB), linker domain (L), SH2 domain (SH2), tail segment domain (TS), and trans-activator domain (TA) are indicated, together with their amino acid boundaries. Dotted box in the wild type sequence delimits the boundaries of the deleted region (30 amino acids) in the long mutant isoform (MUT band a).

A novel heterozygous *STAT1* intron five splice site mutation, c.373-2A>C, abolishing the splice acceptor site of exon 6, was found in a patient with disseminated TB infection (P9). The mutation causes a dramatic reduction in the MaxEnt score for the mutant 3’ splice site (http://hollywood.mit.edu/burgelab/maxent/Xmaxentscan_scoreseq_acc.html). RT-PCR with primers located in exons five and seven of *STAT1* revealed three products in the mutation carrier sample, while only one middle-length band was observed in all samples used as controls (Figure 3A). Sanger sequencing of the respective PCR products confirmed that the middle band (205 bp) corresponds to a wild type variant, the upper band (385 bp) corresponds to aberrant splicing where an upstream splice acceptor site is used, resulting in retention of 174 bp of intronic sequence, and the lower band (115 bp) corresponds to the product in which exon six is skipped (Figure 3B). Thus, we conclude that the mutation *STAT1* c.373-2A>C affects the pre-mRNA splicing *in vivo* (Figure 3C).

### Functional characterization of GBP2 c.1149+14T>C variant

**FIGURE 4:**
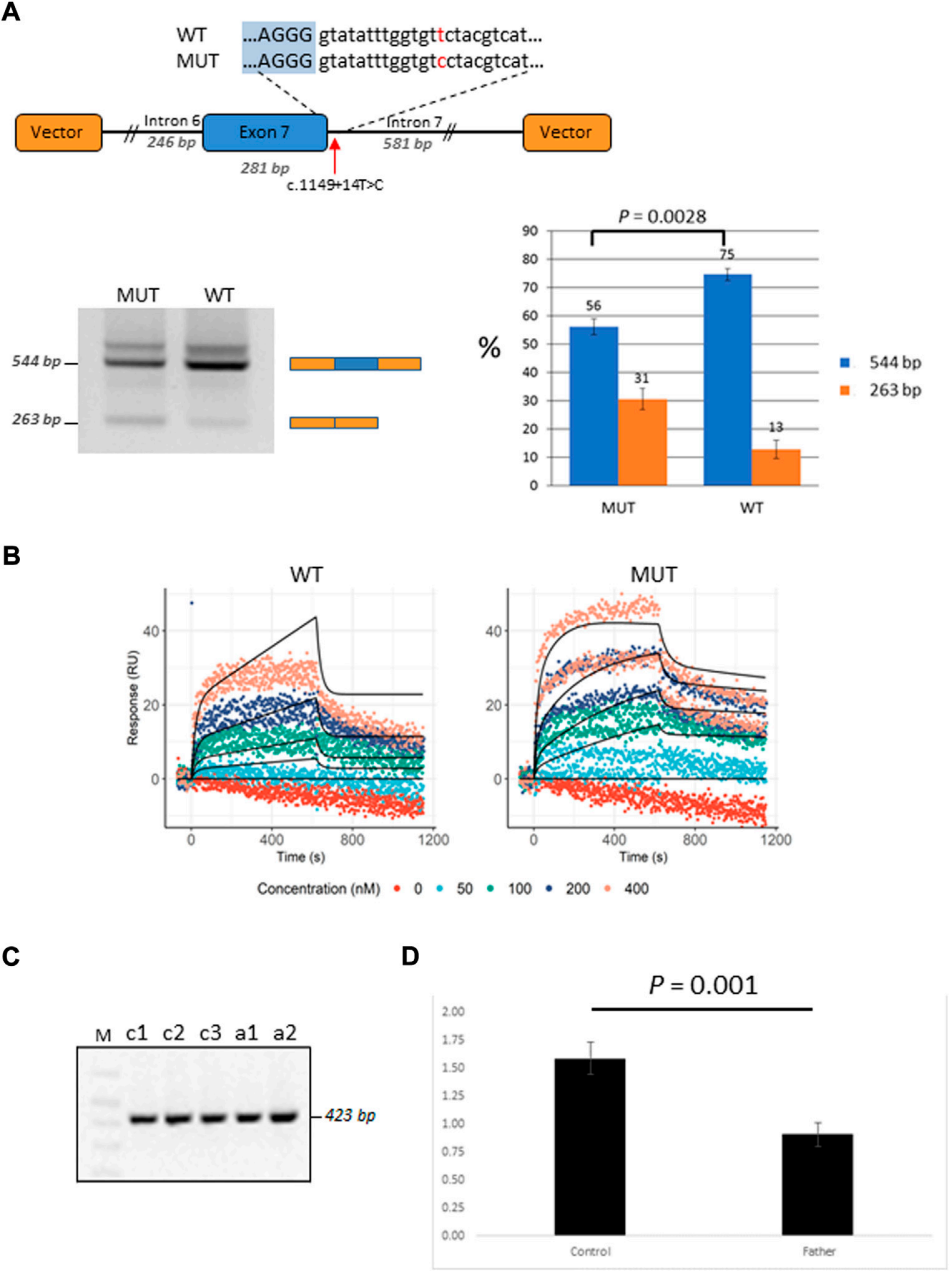
Functional characterization of the *GBP2* intronic variant c.1149+14T>C. **(A)** Minigene analysis of c.1149+14T>C. Illustration of the *GBP2* minigene. The 5′ splice site of exon seven and the downstream sequence covering the c.1149+14T>C variation is enlarged (*top*). RT-PCR analysis of minigene splicing in HepG2 cells. The analysis shows that the minigene carrying the mutant sequence (MUT) has a higher degree of exon seven skipping than the minigene carrying the wild type sequence (WT) (*bottom, left*). Quantification of the exon seven inclusion and exclusion rates (blue and orange columns, respectively). The inclusion/exclusion is quantified on a Fragment Analyzer instrument and represent the intensity of the 544 bp or 263 bp bands over the total intensity in the lane (*bottom, right*). **(B)** SPRi measurements of hnRNP A1 (splicing silencer factor) binding to the WT (the left hand side) and MUT (the right hand side) oligonucleotides. Dots correspond to the raw SPRi measurements while black lines correspond to the model-implied fits. Protein (hnRNP A1) was injected in increasing 2-fold concentrations from 50 to 400 nM shown in color dots. **(C)** RT-PCR analysis of c.1149+14T>C from the mRNA of the mutation carrier (the father of P2; samples: c1 and c2) and three healthy controls (samples: a1, a2 and a3). **(D)** qRT-PCR analysis of total *GBP2* mRNA from whole blood in the carrier (the father of P2) and three healthy controls. *GBP2* transcript levels were normalized by *RPL13A* expression. The values presented are the medians of duplicate determinations ±SD.

Patient P2 was found to be compound heterozygous for two mutations in the *GBP2* gene, namely, a missense mutation c.412G>A/p.(Ala138Thr) and an intronic mutation c.1149+14T>C immediately downstream of the 5′ splice site of exon 7. As RNA was not available from this patient, *in vitro* functional experiments (i.e., mini-gene assay and SPRi analysis) were designed and used for testing the effect of c.1149+14T>C on splicing. In the mini-gene assay, two constructed minigenes, covering exon seven of the *GBP2* gene and carrying either a T (wild type) or a C (mutant) at position c.1149+14T>C, were transfected into HepG2 cells and splice products were subsequently analyzed by RT-PCR (Figure 4A). The analysis showed a slightly decreased exon seven inclusion in HepG2 cells transfected with the MUT minigene as compared to the WT minigene. Quantitative analysis of exon seven inclusion/exclusion showed a statistically significant difference (*p* = 0.0028) between WT and MUT lanes, suggesting that c.1149+14T>C has an effect on splicing (Figure 4A). In particular, the mutation may increase the ability of negative splicing regulatory proteins, such as heterogeneous nuclear ribonucleoproteins (hnRNPs), to bind the pre-mRNA. To investigate this, the kinetics of RNA–protein interactions was analyzed by SPRi using recombinant hnRNP A1 protein and biotin-labeled wild-type or mutant (i.e., harboring c.1149+14T>C) oligonucleotides. The analysis revealed binding of the splicing inhibitory protein hnRNP A1 to oligonucleotides with wild type sequence and the binding was increased for the oligonucleotides carrying mutant sequence (Figure 4B). Thus, the results from the SPRi analysis supports the effect of the c.1149+14T>C variant on the exon seven skipping *via* increased binding of hnRNP A1.

The father of the child (P2) who is a heterozygous carrier of the c.1149+14T>C variant was available for *in vivo* mRNA analysis of *GBP2*. Reverse transcriptase PCR (RT-PCR) analysis of splice variants showed no structural difference in the cDNA between the mutant and control samples for c.1149+14T>C, with only one wild-type band detected (Figure 4C). The absence of alternative transcript variants and inconsistency with the results from *in-vitro* mini-gene assay may be due to nonsense mediated decay (NMD), which recognizes and eliminates abnormal mRNAs making them virtually undetectable. To test this hypothesis, we compared the levels of *GBP2* expression in the carrier of the c.1149+14T>C mutation (i.e., the father of the affected child) with the *GBP2* expression levels in three matched healthy controls. As is shown in Figure 4D, the mutation carrier had significantly lower *GBP2* expression compared to controls (*p* = 0.001). This finding is consistent with the hypothesis of mRNA degradation by NMD and supports the impact of the intronic mutation c.1149+14T>C in *GBP2* on alternative splicing.

### Protein structure prediction

Swiss ExPASy (Expert Protein Analysis System) Molecular Biology Server was used to predict the effects of identified mutations in *HEATR3*, *STAT1* and *GBP2* genes on the protein structure. The *de novo* deletion (-AA) in the third exon of *HEATR3* is a frameshift mutation predicted to cause a severely truncated non-functional HEATR3 protein (Supplementary Figure S2). The skipping of exon six from the *STAT1* gene transcript leads to an in-frame deletion of 90 nucleotides and the removal of 30 amino acids from a stretch linking the N-Terminal to the Coiled-Coil domain of STAT1, while the retention of a 174 bp intronic fragment leads to an out-of-frame change and generates a premature termination codon at position 191 (Figure 3D).

**FIGURE 5:**
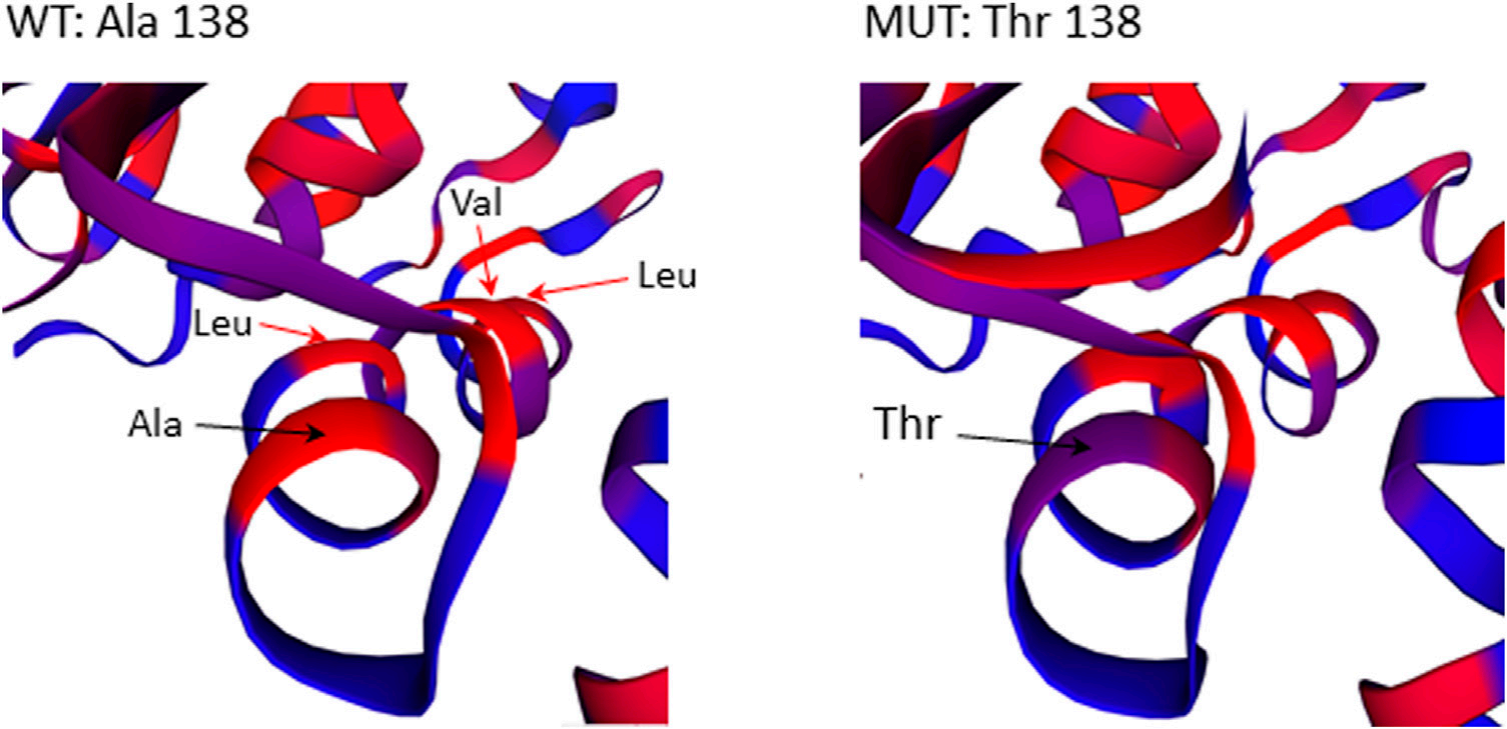
Close-up view of residues surrounding Ala138Thr in the 3D structure of the GBP2 protein. Substitution of Ala with more hydrophilic Thr reduces the stretch of hydrophobicity along one side of the α-helix.

The missense mutation c.412G>A/p.(Ala138Thr) in *GBP2* gene is located in an essential functional domain and was predicted to be deleterious by four out of five *in silico* tools used (Table 2). Noteworthy, the alanine (Ala) at position 138 is one of four hydrophobic residues located along one side of the α-helix (Figure 5). While the structural analysis revealed no essential differences between the wild-type and mutant models, the substitution of Ala to the more hydrophilic threonine (Thr) leads to the reduction in hydrophobicity of the whole helix from -0.68 to -0.92 (QQAMDQLHYV vs*.* QQTMDQLHYV, http://www.gravy-calculator.de), that may affect the stability of the protein. Indeed, the predicted folding free energy change determined using the I-Mutant2.0 software was negative (DDG = -1.64) with a sufficient reliability (RI) of 7.0, indicating a destabilizing effect of the above substitution on the protein structure and possibly function.

### Identification of an oligogenic etiology in patients with mycobacteriosis

**FIGURE 6:**
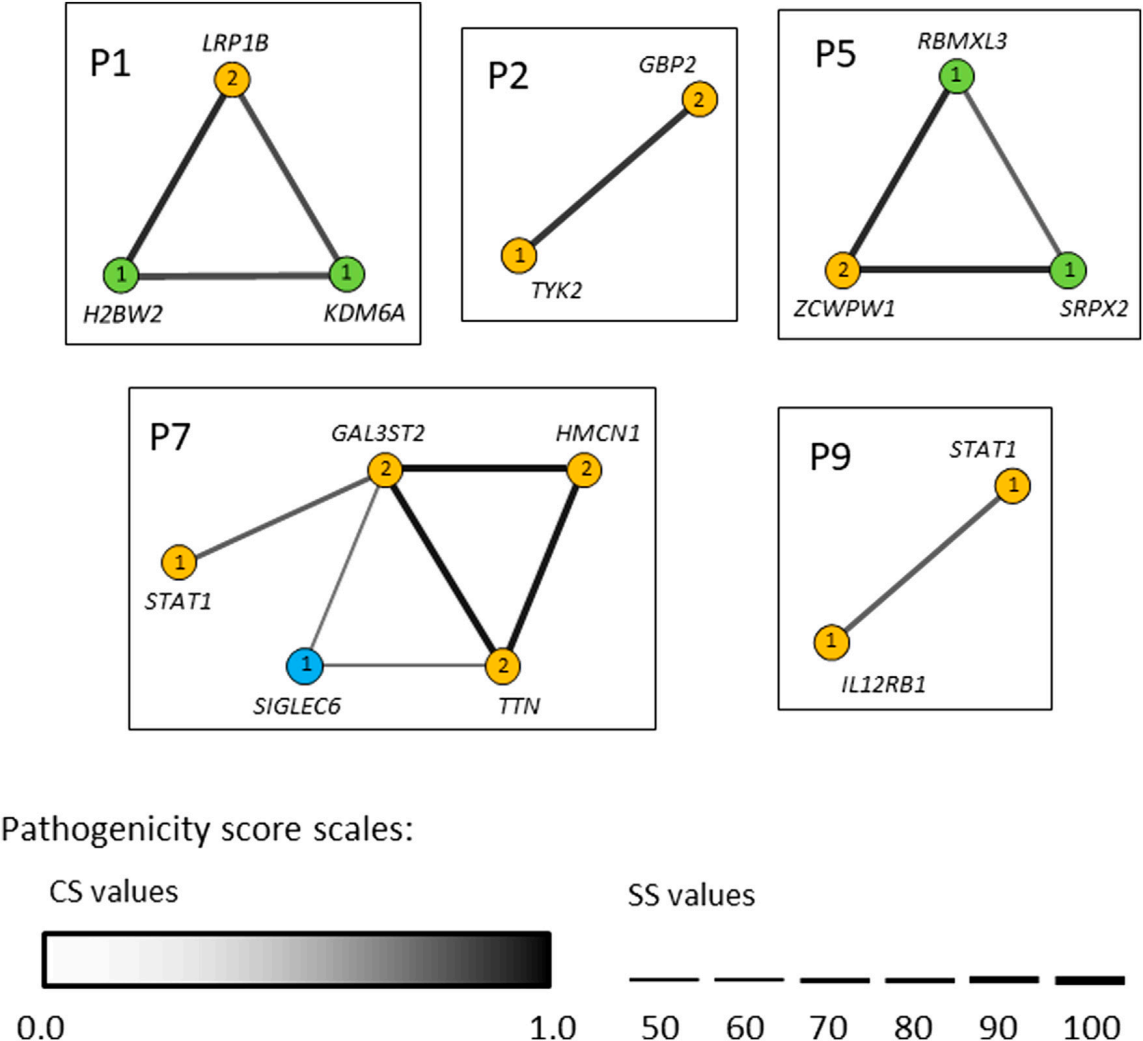
Gene networks showing the epistatic interactions between the genes in which rare variants have been identified. Nodes are individual genes and edges represent pair-wise interactions between genes/variants. Numbers inside the nodes denote number of variants. Yellow, blue and light-green nodes represent heterozygous, homozygous and hemizygous variants, respectively. Edge color intensity is proportional to the pathogenicity classification score (CS), whereas edge thickness is proportional to the pathogenicity support score (SS) for a combination of variants. In case two variants within one gene (i.e., compound heterozygotes), the highest pathogenicity scores computed for variant combinations are depicted. Only disease-causing variant combinations/networks (cutoff >0.532 for CS and ≥50% for SS) are illustrated.

In seven out of nine patients investigated (P1, P2, P3, P5, P6, P7 and P9) the identified variants occurred concomitantly in two or more different candidate genes (Tables 2 and 3). We next used the ORVAL platform to explore their potential digenic or oligogenic impact (i.e., epistatic interactions) in increased predisposition to mycobacterial disease. The analysis identified five patients with at least one pathogenic combination (cutoff >0.532 for CS and ≥50% for SS) between genes (Figure 6). In particular, the variant combinations in the gene pairs *H2BW2*-*LRP1B*, *H2BW2*-*KDM6A* (both in P1), *GBP2*-*TYK2* (in P2), *SRPX2*-*ZCWPW1* and *ZCWPW1*-*RBMXL3* (both in P5) were predicted as disease-causing candidates with 95% confidence, while the variant combinations in the gene pairs *GAL3ST2*-*HMCN*1, *GAL3ST2*-*TTN* and *HMCN1*-*TTN* (all in P7) were within the 99% confidence zone for pathogenicity.

## Discussion

In this study, the genetic etiology of susceptibility to mycobacterial infections was explored in nine pediatric cases by whole-exome sequencing, bioinformatic analysis and functional assessment. As result, several potentially causative rare variants (mostly SNVs) in the MSMD-known and newly discovered genes were identified *via* candidate gene (singleton WES) and child-parents trio approaches.

Using singleton WES, 12 heterozygous (monoallelic) mutations in the eight MSMD-causing genes, namely *TYK2*, *ZNFX1*, *IFNGR1*, *IL23R*, *JAK1*, *IL12B*, *STAT1* and *IL12RB,* were detected in six patients. As mutations in *STAT1* and *IFNGR1* are known to be responsible for both AD and AR forms of mycobacteriosis, the heterozygous variants identified in these genes (*STAT1* c.373–2A>C and *IFNGR1* c.40G>A/p.(Val14Met) are the most promising causal candidates in this study.

The novel splice-site mutation (c.373-2A>C) reported here in the gene *STAT1* in a patient with disseminated TB leads to both partial intron five retention and exon six skipping. The retention of the intronic fragment leads to a frameshift, resulting in early termination of translation and a nonfunctional protein. The in-frame exon skipping event causes a deletion in the protein which would lead to the loss of connection between the N-Terminal and Coiled-Coil domains, which are known to play a role in the dimerization and DNA binding (Mertens et al., 2006). Variants with similar molecular outcomes have been reported previously in several cases with mycobacterial infections (Kong et al., 2010; Kristensen et al., 2011; Vairo et al., 2011; Sakata et al., 2020). These however were in biallelic homozygous or monoallelic compound heterozygous states and were inherited according to an AR mode, while in our case the finding of only a single monoallelic variant is more consistent with an AD inheritance pattern. A simple haploinsufficiency at the *STAT1* locus may provide an explanation for our case, as the dosage of normal STAT1 protein generated by the single wild-type allele could be insufficient for complete protection against the highly virulent *M. tuberculosis* infection in this unprotected (i.e., BCG-unvaccinated) infant. Alternatively, a compound combination with an additional monoallelic variant located in a deep intronic or regulatory region of *STAT1* not captured by our analysis may be casual. This second scenario may also be a cause for BCGitis in five patients with mutations in the *TYK2*, *IL23R*, *JAK1*, *IL12B* and *IL12RB* genes responsible for AR MSMD. Further genetic and functional studies should be carried out to evaluate the exact biological effects and inheritance pattern of the identified mutants. Regarding *IFNGR1*, although the identified variant *IFNGR1* c.40G>A/p.(Val14Met) has a relatively high allele population frequency (0.002–0.003) in Europe, it was proven in previous studies to be damaging and was enriched in patients with eczema herpeticum (ADEH+)—a rare and serious skin infection caused by the herpes simplex virus (Gao et al., 2015).

In a trio approach, 25 variants in 15 unknown genes were bioinformatically prioritized. We then critically revised these genes by examining their expressions in immune related tissues. As a result, genes *GBP2*, *HEATR3*, *PPP1R9B* and *KDM6A*, showing elevated expressions in whole blood, spleen or EBV-transformed lymphocytes, were selected as top-ranked candidates for susceptibility to BCG infection in this study. Of these new candidate genes, *GBP2* displays the closest biological relationship with the known MSMD-causing genes. The encoded by *GBP2* protein (Guanylate Binding Protein 2) is a member of interferon-gamma induced guanylate-binding proteins (Ramsauer et al., 2007; Yan et al., 2016). Following induction, GBPs confer protective immunity against a variety of microbial pathogens (Kim et al., 2011; Praefcke, 2018; Tretina et al., 2019). Previous experimental studies using animal models and also functional gene-expression studies demonstrated its important role in the protection against mycobacteria (Berry et al., 2010; Kim et al., 2011; Marquis et al., 2011; Tretina et al., 2019; Perumal et al., 2021). In this study, two compound heterozygous mutations in *GBP2* gene (c.412G>A/p.(Ala138Thr) and c.1149+14T>C) in a patient with BCG infection (P2) were identified. The maternally inherited missense mutation p.Ala138Thr was predicted to be deleterious by the majority of *in silico* tools, possibly having an impact on the protein folding and stability. Functional analysis of the second, paternally inherited intronic mutation c.1149+14T>C, demonstrated that this variant is likely to affect splicing of the GBP2 mRNA transcript, leading to decreased levels of functional transcript.

A recently discovered MSMD-causing gene *SPPL2A* was an outlier compared to the other MSMD-causing genes in our gene networking and connectome server analyses (Figure 2). Likewise, prioritized novel genes in the present study, although not directly linked to the core MSMD-causing genes, may also be involved in the disease pathways. Indeed, *HEATR*3 is a component of the NOD2-mediated NF-kappaB signaling, which plays a crucial role in the control of *Mycobacterium* within host macrophages (Deng and Xie, 2012; Zhang et al., 2013). Here, we identified a deletion frameshift mutation c.395_396delAA/p.(Lys132Argfs*3) in *HEATR3* in a patient with BCG-related complications*.* The variant introduces an early premature termination codon, which could either be associated with no protein due to nonsense-mediated mRNA decay or a severely C-terminally truncated protein missing all functional domains. Moreover, the *de novo* status of this variant in our patient increases it is likelihood for being a real cause of BCGitis. Also, undescribed *de novo* disruptive in-frame deletion was found in *PPP1R9B*, which encodes a scaffolding protein involved in multiple signaling pathways and cytoskeletal rearrangement in NK cells (Meng et al., 2009).

Other genes in this study, including *TTN* and *LRP1B*, are secondary candidates, because of their insignificant expression in immune cells, although their impact cannot be completely excluded.

With the increasing use of next generation sequencing, it has become clear that oligogenic inheritance is more common than previously thought for hereditary disorders, including PIDs (Rigaud et al., 2011; Chong et al., 2015; de Valles-Ibáñez et al., 2018). Here, we identified five patients with potential oligogenic inheritance. Noteworthy, two of these cases carried trans heterozygous (monoallelic) variants in two genes belonging to the IL-12/IL-23/IFN-γ axis, namely, *IL12RB1* and *STAT1* in the patient P9, and *TYK2* and *GBP2* (a novel candidate-gene) in the patient P2. Since *IL12RB1*, *STAT1* and *TYK2* separately are known to cause autosomal recessive MSMD and *GBP2* is a novel plausible candidate for MSMD, reduced activity of both proteins in the same close interacting network is a likely disease model. This disease mechanism, termed ‘synergistic heterozygosity’ (Vockley et al., 2000), could also explain differences in the penetrance of mycobacterial disease in some families and the above-mentioned inconsistency with previously published results in the inheritance of *STAT1* c.373–2A>C*.* Also, *H2BW2* (a histone protein) and *KDM6A* (a histone H3 demethylase protein) belong to the same chromatin remodeling pathway regulating gene expression. Although experimental validation of the oligogenic model still needs to be conducted, our data provide insight into the complex disease-causing mode of mycobacterial disease and suggest the combined effect of mutations in IL-12/IL-23/IFN-γ mediated and other pathways genes as an important mechanism in the susceptibility to mycobacterial infections.

## Conclusion

The analysis presented here expand the spectrum of genetic variation underlying mycobacterial infections in children. Indeed, we identified 12 heterozygous variants, two of which were novel, in eight known MSMD-causing genes and found pathogenic or likely-pathogenic variants in 15 new candidate genes. Besides this, our study provides insights into a potential oligogenic disease-causing mode for conferring susceptibility to mycobacterial infections. The obtained results rise several topics for further research, including methodological application of WES to identify novel genes for susceptibility to mycobacterial and other infections, experimental and population-based validations of the identified variants and genes, role of oligogenic inheritance in susceptibility to infectious diseases, haploinsufficiency of *STAT1* and other MSMD causing genes, relevance of intronic variants, compound heterozygous inheritance of MSMD, *etc.* Also, CNV and epigenetic modifications that were not tested are assumed for causality in this study and in general. Revealing novel genes and molecular mechanisms underlying mycobacterial infections in children might facilitate the development of therapeutic strategies to prevent and treat mycobacterial disease.

## Data Availability

Variants with functional assessments in this study are deposited in the ClinVar repository (https://www.ncbi.nlm.nih.gov/clinvar/; submission SUB12127021, accession numbers SCV002581932 - SCV002581934). All other relevant data supporting conclusions are included in the article/Supplementary Material or available by request.
