# Supplementary material for: Candidate genes and sequence variants for susceptibility to mycobacterial infection identified by whole-exome sequencing
Source: Front Genet. 2022 Oct 20;13:969895. doi: 10.3389/fgene.2022.969895 (PMC9632272; doi:10.3389/fgene.2022.969895)
Supplement: Supplementary file 1 [file Table1.DOCX]

**Supplementary Table S1.** Summary of whole-exome sequencing data (Important QC values from NGSD)

| **Sample** | **Total number of reads at Q0** | **Read length** | **Reads at Q20 (%)** | **Reads at Q30 (%)** | **Average sequencing depth** | **Target region 10x (%)** | **Target region 20x (%)** | **Total number of variants in the target region** |
| --- | --- | --- | --- | --- | --- | --- | --- | --- |
| P7-child | 109184430 | 35-151 | 99.04 | 83.57 | 134.13 | 98.37 | 97.41 | 40667 |
| P7-mother | 93373950 | 35-151 | 99.20 | 84.03 | 120.39 | 98.54 | 98.14 | 40304 |
| P7-father | 103024102 | 35-151 | 99.46 | 84.06 | 129.92 | 98.68 | 98.29 | 40232 |
| P2-child | 79052092 | 35-151 | 99.08 | 80.74 | 96.68 | 98.43 | 97.53 | 40238 |
| P2-mother | 92159484 | 35-151 | 99.42 | 81.40 | 105.54 | 98.56 | 98.04 | 40156 |
| P2-father | 97377368 | 35-151 | 99.45 | 80.68 | 112.58 | 98.75 | 98.16 | 40562 |
| P6-child | 96061898 | 35-151 | 99.32 | 82.15 | 134.52 | 97.54 | 94.69 | 39796 |
| P6-father | 103246428 | 35-151 | 98.96 | 82.50 | 137.38 | 98.42 | 97.43 | 39732 |
| P6-mother | 121992418 | 35-151 | 99.27 | 82.82 | 150.06 | 95.30 | 90.57 | 39009 |
| P5-child | 89598730 | 35-151 | 98.84 | 83.82 | 114.56 | 98.21 | 97.58 | 39950 |
| P5-mother | 229127222 | 35-151 | 98.63 | 83.31 | 244.89 | 98.68 | 98.54 | 40893 |
| P5-father | 75518758 | 35-151 | 98.34 | 83.11 | 96.75 | 98.33 | 96.60 | 39436 |
| P3-child | 81400678 | 35-151 | 98.20 | 84.79 | 98.43 | 97.76 | 95.05 | 39926 |
| P3-mother | 107590406 | 35-151 | 98.58 | 85.33 | 126.88 | 98.37 | 97.35 | 40693 |
| P3-father | 104342284 | 35-151 | 98.42 | 85.93 | 126.89 | 98.57 | 98.10 | 39892 |
| P4-child | 86083234 | 35-151 | 98.14 | 82.02 | 117.80 | 96.94 | 92.81 | 39508 |
| P4-father | 113472864 | 35-151 | 98.11 | 83.24 | 147.36 | 98.73 | 98.45 | 39475 |
| P4-mother | 96995842 | 35-151 | 98.13 | 82.91 | 125.77 | 98.42 | 98.02 | 40098 |
| P1-child | 98607514 | 35-151 | 98.37 | 82.21 | 132.28 | 98.62 | 98.24 | 40252 |
| P1-mother | 103807482 | 35-151 | 98.21 | 81.92 | 141.71 | 98.71 | 98.45 | 39765 |
| P1-father | 97914688 | 35-151 | 98.47 | 81.56 | 129.07 | 98.58 | 98.09 | 39999 |
| P8 | 79386442 | 35-151 | 98.41 | 81.26 | 111.01 | 98.27 | 97.71 | 39897 |
| P9 | 91302102 | 35-151 | 97.82 | 82.46 | 119.21 | 98.35 | 97.88 | 39719 |

**Q0:** without applying read quality step or as it was given by the sequencer; **Q20** and **Q30:** after applying the quality steps

**Supplementary Table S2.** The results of the trio-based WES analysis and variants filtering for seven patients

| **Step** | **Trio P7** | **Trio P2** | **Trio P6** | **Trio P5** | **Trio P3** | **Trio P4** | **Trio P1** |
| --- | --- | --- | --- | --- | --- | --- | --- |
| Initial number of variants | 41943 | 41616 | 41139 | 41134 | 41223 | 40864 | 41338 |
| After QC: low MQM, low QUAL ^a^ | 38617 | 38185 | 37931 | 37891 | 37847 | 37723 | 38232 |
| MAF < 1%^a^ | 2709 | 2753 | 2742 | 2523 | 2625 | 2603 | 2516 |
| Listed in the in-house database (ihdb) < 20 ^a^ | 1484 | 1301 | 1247 | 1060 | 1040 | 1028 | 1074 |
| Compound heterozygous recessive ^b^ | 65 | 63 | 73 | 54 | 60 | 28 | 46 |
| Homozygous recessive ^b^ | 3 | 1 | 3 | 0 | 3 | 3 | 3 |
| X-linked hemizygous ^b^ | NA | NA | NA | 14 | NA | NA | 18 |
| *De novo* (sporadic) variants ^b^ | 2 | 3 | 3 | 3 | 2 | 1 | 0 |

^a^Automatic filtering in GSVar

^b^After manual inspection in IGV

NA – not applicable

**Supplementary Table S3.** The results of the singleton WES analysis and variants filtering for nine patients

| **Step** | **P7** | **P2** | **P6** | **P5** | **P3** | **P4** | **P1** | **P8** | **P9** |
| --- | --- | --- | --- | --- | --- | --- | --- | --- | --- |
| Initial number of variants | 66864 | 65643 | 64739 | 65553 | 65456 | 64445 | 66073 | 65328 | 65517 |
| After QC: low MQM, low QUAL ^a^ | 62453 | 61052 | 60447 | 61195 | 60793 | 60021 | 61860 | 60928 | 61195 |
| MAF < 1% ^a^ | 3133 | 2884 | 2915 | 2756 | 2840 | 2893 | 2796 | 2688 | 2809 |
| Listed in our in-house database (ihdb) < 20 ^a^ | 1796 | 1668 | 1747 | 1473 | 1336 | 1403 | 1429 | 1381 | 1356 |
| Filtered by MSMD-causing genes ^a^ | 1 | 2 | 3 | 0 | 3 | 0 | 0 | 1 | 2 |
| Filtered by MSMD-causing genes ^b^ | 1 | 2 | 3 | 0 | 3 | 0 | 0 | 1 | 2 |

^a^Automatic filtering in GSVar

^b^After manual inspection in IGV

**Supplementary Table S4.** Rare variants (MAF < 0.01) identified in RPP genes (*TLR1, TlR2, TLR3, TLR4, TLR5, TLR6, TLR7, TLR8, TLR9, TLR10, CD14, NOD2, NLRP3, FCRLB, CLEC4E, CLEC7A, MRC1, CD209, MSR1, SCARB1, MARCO, CD36, STING1, AIM2, CGAS,* and *MBL2*)

| Patient | Gene | Nucleotide change (transcript GRCh37/hg19) | Protein variant | Type of mutation | rs ID | Zygocity | GnomAD |
| --- | --- | --- | --- | --- | --- | --- | --- |
| P7 | *NOD2* | NM_022162.3:c.460G>A | p.(Asp154Asn) | Missense | rs146054564 | het | 0.0006916 |
|  | *MARCO* | NC_000002.11:g.119732161G>T (NM_006770.4:c.613+20G>T) | p.(=) | Intron and splice region | rs367715327 | het | 0.0007448 |
| P2 | *CD209* | NC_000019.9:g.7810011C>T (NM_021155.4:c.749-33C>T) | p.(=) | Intron | rs113836492 | het | 0.006764 |
|  | *MSR1* | NC_000008.10:g.15997293A>T  (NM_138715.3:c.1033+3774A>T) | p.(=) | Intron | rs1197123205 | het | 0.003158 |
|  | *SCARB1* | NC_000012.11:g.125296386G>A  (NM_005505.5:c.726+30G>A) | p.(=) | Intron | rs144347463 | het | 0.003119 |
| P6 | *NOD2* | NM_022162.3:c.2470G>A | p.(Asp824Asn) | Missense | rs61755272 | het | 0.0002547 |
| P5 | *TLR9* | NM_017442.4:c.2988C>T | p.(=) | Synonymous | rs1172635789 | het | 0.000004569 |
|  | *CD36* | NC_000007.13:g.80299255C>T  (NM_000072.3:c.749-14C>T) | p.(=) | Intron and splice region | rs200439592 | het | 0.001477 |
| P1 | *TLR1* | NM_003263.4:c.960T>C | p.(=) | Synonymous | rs200631178 | het | 0.00009563 |
| P8 | *TLR7* | NM_016562.4:c.1343C>T | p.(Ala448Val) | Missense | rs5743781 | het | 0.003006 |
|  | *MSR1* | NC_000008.10:g.16026383A>G  (NM_002445.3:c.218-4A>G) | p.(=) | Intron and splice region | rs189127302 | het | 0.0006619 |
|  | *MARCO* | NC_000002.11:g.119749959G>A  (NM_006770.4:c.1208-49G>A) | p.(=) | Intron | rs374645210 | het | 0.0001743 |

**Supplementary Table S5.** Sequencing quality parameters of the prioritized variants listed in Tables 2 and 3

| Trio/ patient | Variant | Gene | Approach used | QUAL | DP | MQM |
| --- | --- | --- | --- | --- | --- | --- |
| P1 | NM_018557.3:c.11227G>A | *LRP1B* | Trio | 2599 | 120 | 60 |
|  | NM_018557.3:c.1907G>A | *LRP1B* | Trio | 2537 | 112 | 60 |
|  | NM_021140.4:c.1402T>C | *KDM6A* | Trio | 1179 | 29 | 60 |
|  | NM_001164416.3:c.368T>G | *H2BW2* | Trio | 3771 | 66 | 60 |
| P2 | NC_000001.10:g.89579685A>G (NM_004120.5:c.1149+14T>C) | *GBP2* | Trio | 2756 | 128 | 60 |
|  | NM_004120.5:c.412G>A | *GBP2* | Trio | 996 | 50 | 60 |
|  | NC_000003.11:g.49753164C>T (NM_022064.5:c.3150+17C>T) | *RNF123* | Trio | 959 | 120 | 60 |
|  | NM_003331.5:c.2441C>T | *TYK2* | Single-case | 1471 | 108 | 60 |
|  | NC_000020.10:g.47877143G>A (NM_021035.3:c.2302-29C>T) | *ZNFX1* | Single-case | 494 | 47 | 60 |
| P3 | NC_000017.10:g.48227115_48227123del (NM_032595.5:c.759_767del | *PPP1R9B* | Trio | 1448 | 185 | 60 |
|  | NM_000416.3:c.40G>A | *IFNGR1* | Single-case | 1258 | 132 | 60 |
|  | NM_144701.3:c.257G>A | *IL23R* | Single-case | 399 | 42 | 60 |
|  | NM_002227.4:c.414C>T | *JAK1* | Single-case | 1004 | 86 | 60 |
| P4 | NM_133378.4:c.77167C>T | *TTN* | Trio | 4837 | 179 | 60 |
|  | NM_133378.4:c.68129G>A | *TTN* | Trio | 4707 | 241 | 60 |
| P5 | NM_017984.6:c.1049A>G | *ZCWPW1* | Trio | 2247 | 70 | 60 |
|  | NM_017984.6:c.314A>G | *ZCWPW1* | Trio | 1128 | 54 | 60 |
|  | NM_014467.3:c.517G>A | *SRPX2* | Trio | 4909 | 47 | 60 |
|  | NM_001145346.2:c.128G>A | *RBMXL3* | Trio | 8013 | 91 | 60 |
| P6 | NM_002187.3:c.877A>G | *IL12B* | Single-case | 3060 | 125 | 60 |
|  | NC_000005.9:g.158750351A>G (NM_002187.3:c.89-14T>C) | *IL12B* | Single-case | 2048 | 100 | 60 |
|  | NM_182922.4:c.395_396delAA | *HEATR3* | Trio | 270 | 37 | 60 |
|  | NM_002177.3:c.58G>A | *IFNW1* | Trio | 5846 | 130 | 60 |
|  | NM_003331.5:c.157G>A | *TYK2* | Single-case | 1045 | 123 | 60 |
| P7 | NM_133378.4:c.97070A>C | *TTN* | Trio | 4691 | 224 | 60 |
|  | NM_133378.4:c.75185A>C | *TTN* | Trio | 5588 | 227 | 60 |
|  | NC_000002.11:g.242716400G>A (NM_022134.3:c.29+1G>A) | *GAL3ST2* | Trio | 1627 | 94 | 60 |
|  | NM_022134.3:c.458A>G | *GAL3ST2* | Trio | 3393 | 187 | 60 |
|  | NM_031935.3:c.109G>A | *HMCN1* | Trio | 3440 | 145 | 60 |
|  | NM_031935.3:c.4586A>G | *HMCN1* | Trio | 1981 | 53 | 60 |
|  | NM_001245.7: c.292G>T | *SIGLEC6* | Trio | 9381 | 199 | 59 |
|  | NC_000002.11:g.191864302A>G (NM_007315.4:c.541+50T>C) | *STAT1* | Single-case | 519 | 50 | 60 |
| P8 | NM_002187.3:c.961G>A | *IL12B* | Single-case | 927 | 87 | 60 |
| P9 | NC_000002.11:g.191865891T>G (NM_007315.4:c.373-2A>C) | *STAT1* | Single-case | 446 | 47 | 60 |
|  | NC_000019.9:g.18173093G>A (NM_005535.3:c.1619-6C>T) | *IL12RB1* | Single-case | 684 | 44 | 60 |

**Supplementary Table S6.** Prioritization of candidate genes using tissue-specific gene expression profiles from GTEx database. Gene expression is given in TPM values. New candidate genes with the higher expression levels in whole blood or immune-related cells (i.e., spleen or EBV-transformed lymphocytes) were given the highest priority in this study (marked in red)

| **Gene** | **Sex** | **Min (Tissue)** | **Max (Tissue)** | **Whole Blood** | **Spleen** | **EBV-transformed lymphocytes** |
| --- | --- | --- | --- | --- | --- | --- |
| New candidate genes | | | | | | |
| *GBP2* | both | 1.069 (Brain - Cerebellar Hemisphere) | 106 (Whole Blood) | 106 | 47.81 | 28.82 |
| *HEATR3* | both | 4.440 (Brain Amygdala) | 23.86 (Spleen) | 5.367 | 23.83 | 16.90 |
| *TTN* | both | 0.09455 (Brain - Amygdala) | 358.5 (Muscle - Skeletal) | 0.2851 | 0.7224 | 0.8848 |
| *GAL3ST2* | both | 0.01935 (Heart - Left Ventricle) | 5.010 (Colon - Transverse) | 0.02848 | 0.07242 | 0.4035 |
| *HMCN1* | both | 0.01722 (Whole Blood) | 38.19 (Artery - Aorta) | 0.01722 | 2.234 | 0.02125 |
| *ZCWPW1* | both | 2.139 (Pancreas) | 144 (Testis) | 2.918 | 9.176 | 4.976 |
| *LRP1B* | both | 0.002240 (Whole Blood) | 4.729 (Brain - Cerebellum) | 0.002240 | 0.01032 | 0.003770 |
| *PPP1R9B* | both | 8.225 (Liver) | 176.8 (Brain - Cerebellar Hemisphere) | 55.84 | 96.15 | 94.40 |
| *SRPX2* | males | 0.02507 (EBV-transformed lymphocyte) | 39.57 (Adipose - Subcutaneous) | 0.02525 | 12.21 | 0.02507 |
| *RBMXL3* | males | 0.000 (Whole Blood) | 6.781 (Testis) | 0.000 | 0.000 | 0.000 |
| *KDM6A* | males | 2.140 (Brain - Putamen - basal ganglia) | 23.43 (Testis) | 6.043 | 11.25 | 13.32 |
| *H2BFM* | males | 0.000 (Whole Blood) | 2.389 (Testis) | 0.000 | 0.000 | 0.000 |
| *SIGLEC6* | both | 0.000 (Pituitary) | 2.137 (Small Intestine - Terminal Ileum) | 0.09785 | 0.5891 | 0.2517 |
| *IFNW1* | both | 0.000 (Whole Blood) | 0.1939 (Testis) | 0.000 | 0.000 | 0.02793 |
| *RNF123* | both | 4.947 (Brain - Substantia nigra) | 74.03 (Muscle - Skeletal) | 15.60 | 18.78 | 12.21 |
| Known MSMD-causing genes | | | | | | |
| *IL12RB1* | both | 0.04878 (Cells - Cultured fibroblasts) | 17.52 (Spleen) | 9.762 | 17.52 | 16.54 |
| *IL12B* | both | 0.000 (Vagina) | 1.003 (EBV-transformed lymphocytes) | 0.01401 | 0.09785 | 1.003 |
| *IL12RB2* | both | 0.04693 (Brain - Putamen - basal ganglia) | 5.900 (EBV-transformed lymphocytes) | 0.7386 | 1.068 | 5.900 |
| *ISG15* | both | 5.409 (Pancreas) | 611.4 (EBV-transformed lymphocytes) | 28.80 | 57.44 | 611.4 |
| *SPPL2A* | both | 3.195 (Brain - Putamen - basal ganglia) | 36.53 (Muscle - Skeletal) | 8.613 | 17.43 | 19.01 |
| *IRF8* | both | 0.02261 (Cells - Cultured fibroblasts) | 136 (EBV-transformed lymphocytes) | 18.10 | 107.3 | 136 |
| *TYK2* | both | 12.21 (Brain - Putamen - basal ganglia) | 100.4 (EBV-transformed lymphocytes) | 78.64 | 95.00 | 100.4 |
| *FNGR1* | both | 21.32 (Pancreas) | 268.1 (Lung) | 198.3 | 207.8 | 108.8 |
| *IFNGR2* | both | 10.18 (Pancreas) | 117.1 (Whole Blood) | 117.1 | 70.65 | 65.31 |
| *STAT1* | both | 12.14 (Heart - Left Ventricle) | 501.4 (EBV-transformed lymphocytes) | 33.23 | 61.68 | 501.4 |
| *IKBKG* | males | 1.361 (Brain - Putamen - basal ganglia) | 7.457 (Cells - Cultured fibroblasts) | 7.056 | 7.210 | 6.024 |
| *CYBB* | males | 0.02660 (Cells - Cultured fibroblasts) | 92.78 (Whole Blood) | 92.78 | 83.89 | 55.25 |
| *JAK1* | both | 18.68 (Brain - Hippocampus) | 139.9 (Cells - Cultured fibroblasts) | 62.14 | 95.08 | 51.36 |
| *RORC* | both | 0.02521 (Cells - Cultured fibroblasts) | 47.69 (Muscle - Skeletal) | 0.6223 | 1.213 | 0.02784 |
| *IL23R* | both | 000 (Uterus) | 2.468 (EBV-transformed lymphocytes) | 0.06025 | 0.01636 | 2.468 |
| *ZNFX1* | both | 3.969 (Pancreas) | 35.54 (EBV-transformed lymphocytes) | 21.91 | 24.68 | 35.54 |
| *TBX21* | both | 0.07381 (Brain - Anterior cingulate cortex) | 18.48 (Spleen) | 14.43 | 18.48 | 15.97 |
| *IFNG* | both | 000 (Uterus) | 1.075 (Whole Blood) | 1.075 | 0.9169 | 0.8554 |
| *USP18* | both | 0.4677 (Whole Blood) | 45.30 (EBV-transformed lymphocytes) | 0.4677 | 4.169 | 45.30 |

**Supplementary Table S7.** Estimates of the probability of true-positive matches between genes and disease using RD-Match service

| Gene | Recessivity | Number of patients with a mutated gene/total number of patients | *p*-value (Option: Input exact number below) | *p*-value (Option: Estimate with HPO parameter - Immunodeficiency) |
| --- | --- | --- | --- | --- |
| New candidate genes | | | | |
| *TTN* | compound het | 2/7 | 1.0 | 1.0 |
| *LRP1B* | compound het | 1/7 | 1.0 | 1.0 |
| *KDM6A* | recessive | 1/7 | 0.570206003203 | 0.999085255088 |
| *H2BW2* | recessive | 1/7 | NA | NA |
| *GBP2* | compound het | 1/7 | 0.999698203046 | 1.0 |
| *RNF123* | dominant | 1/7 | 1.0 | 1.0 |
| *PPP1R9B* | dominant | 1/7 | 1.0 | 1.0 |
| *ZCWPW1* | compound het | 1/7 | 0.999995766088 | 1.0 |
| *SRPX2* | recessive | 1/7 | 0.464342828892 | 0.994329328256 |
| *RBMXL3* | recessive | 1/7 | NA | NA |
| *IL12B* | compound het | 1/7 | 0.876678705032 | 0.999999970583 |
| *HEATR3* | dominant | 1/7 | 1.0 | 1.0 |
| *IFNW1* | recessive | 1/7 | 0.346225013799 | 0.970440871172 |
| *GAL3ST2* | compound het | 1/7 | 0.99999992574 | 1.0 |
| *HMCN1* | compound het | 1/7 | 1.0 | 1.0 |
| *SIGLEC6* | recessive | 1/7 | 0.605673072217 | 0.999551892832 |
| Known MSMD-causing genes* | | | | |
| *IL12RB1* | recessive | 1/7 | 0.699416780692 | 0.999952732251 |
| *IL12B* | recessive | 1/7 | 0.309146095505 | 0.953312558066 |
| *IL23R* | recessive | 1/7 | 0.412678990419 | 0.987839015575 |
| *IL12RB2* | recessive | 1/7 | 0.696525553202 | 0.999948830412 |
| *SPPL2A* | recessive | 1/7 | 0.287748930847 | 0.939888155696 |
| *IRF8* | recessive | 1/7 | 0.503706647083 | 0.996987161869 |
| *IRF8* | dominant | 1/7 | 1.0 | 1.0 |
| *IFNGR1* | recessive | 1/7 | 0.418645639085 | 0.988825582678 |
| *IFNGR1* | dominant | 1/7 | 1.0 | 1.0 |
| *IFNGR2* | recessive | 1/7 | 0.325714571306 | 0.961818901543 |
| *IFNGR2* | dominant | 1/7 | 1.0 | 1.0 |
| *STAT1* | recessive | 1/7 | 0.200291762986 | 0.843065395616 |
| *STAT1* | dominant | 1/7 | 1.0 | 1.0 |
| *IKBKG* | recessive | 1/7 | NA | NA |
| *CYBB* | recessive | 1/7 | 0.34007832758 | 0.968057748323 |
| *TYK2* | recessive | 1/7 | 0.877756071758 | 0.999999972646 |
| *JAK1* | recessive | 1/7 | 0.664263893698 | 0.99988181693 |
| *RORC* | recessive | 1/7 | 0.438033973272 | 0.991563368502 |
| *ISG15* | recessive | 1/7 | 0.250218379129 | 0.908008985777 |
| *ZNFX1* | recessive | 1/7 | 0.794667270857 | 0.999952732251 |
| *TBX21* | recessive | 1/7 | 0.393906515403 | 0.953312558066 |
| *IFNG* | recessive | 1/7 | 0.0414419021784 | 0.987839015575 |
| *USP18* | recessive | 1/7 | 0.281431630547 | 0.999948830412 |

*Assuming the total number of patients n =7 and the number of patients with a mutated gene n = 1.
