# Supplementary material for: Candidate genes and sequence variants for susceptibility to mycobacterial infection identified by whole-exome sequencing
Source: Front Genet. 2022 Oct 20;13:969895. doi: 10.3389/fgene.2022.969895 (PMC9632272; doi:10.3389/fgene.2022.969895)
Supplement: Supplementary file 2 [file Image1.pdf]

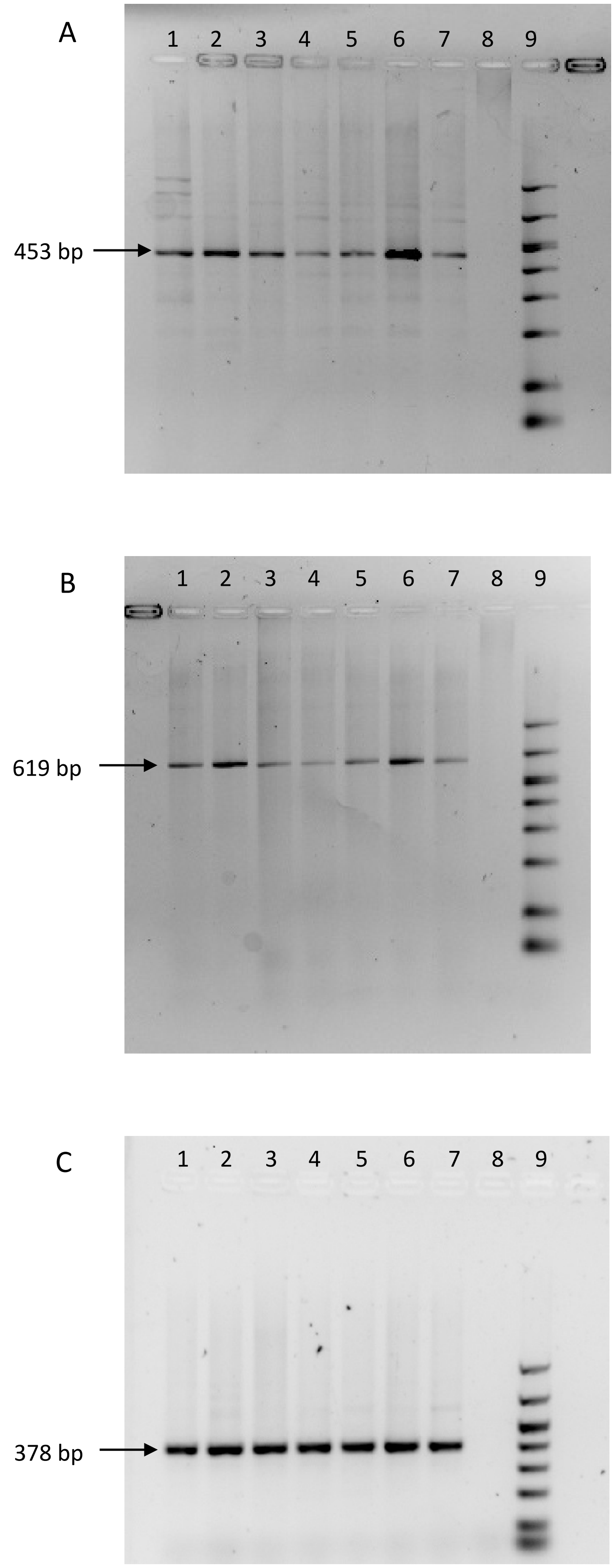

Figure S1. Agarose gel electrophoresis of PCR-amplified cDNA. Loci: (A) *IL12B* c.89-14T>C; (B) *IL12B* c.877A>G; (C) *HEATR3* c.395_396delAA. Line 9: 100-bp (50-700 bp) DNA ladder; line 8: negative PCR control; lines 1, 5-7: healthy paediatric controls; line 2: patient; line 3: patient's father (the carrier of *IL12B* c.89-14T>C); line 4: patient's mother (the carrier of *IL12B* c.877A>G). The specific PCR product is indicated with an arrow.

Figure S2. *De novo* c.395_396delAA variant in the exon 3 of the *HEATR3* gene leading to a frameshift (Lys132fs) and a premature stop codon (as predicted by ExPASy service). The deleted nucleotides are highlighted in yellow.

A)

WT

#	1	2	3	…	122	123	124	125	126	127	128	129	130	131	132	133	134	...	678	679	680
Codon	ATG	GGC	AAG	..//..	GAT	ATC	ATG	ACC	CCT	CTG	GTT	GCG	CTG	CTA	AAA	GAG	TGT	..//..	ACT	TCT	TAA
AA	Met	Gly	Lys	..//..	Asp	Ile	Met	Thr	Pro	Leu	Val	Ala	Leu	Leu	Lys	Glu	Cys	..//..	Thr	Ser	STOP

MUT

#	1	2	3	...	122	123	124	125	126	127	128	129	130	131	132	133	134	...	678	679	680
Codon	ATG	GGC	AAG	..//..	GAT	ATC	ATG	ACC	CCT	CTG	GTT	GCG	CTG	CTA	AGA	GTG	TAG		TTC	TTA	A
AA	Met	Gly	Lys	..//..	Asp	Ile	Met	Thr	Pro	Leu	Val	Ala	Leu	Leu	Arg	Val	STOP				
